# Synthetic Tabular Data Generation Under Horizontal Federated Learning Environments in Acute Myeloid Leukemia: Case-Based Simulation Study

**DOI:** 10.2196/74116

**Published:** 2025-09-29

**Authors:** Imanol Isasa, Mikel Catalina, Gorka Epelde, Naiara Aginako, Andoni Beristain

**Affiliations:** 1 Digital Health & Biomedical Technologies Department Vicomtech Foundation (BRTA) Donostia-San Sebastián Spain; 2 Computer Science and Artificial Intelligence Department University of the Basque Country UPV/EHU Donostia-San Sebastián Spain; 3 eHealth Group Biogipuzkoa Health Research Institute Donostia-San Sebastián Spain

**Keywords:** rare diseases, privacy, machine learning, federated learning, synthetic data generation, leukemia, data fidelity, trade-off

## Abstract

**Background:**

Data scarcity and dispersion pose significant obstacles in biomedical research, particularly when addressing rare diseases. In such scenarios, synthetic data generation (SDG) has emerged as a promising path to mitigate the first issue. Concurrently, federated learning is a machine learning paradigm where multiple nodes collaborate to create a centralized model with knowledge that is distilled from the data in different nodes, but without the need for sharing it. This research explores the combination of SDG and federated learning technologies in the context of acute myeloid leukemia, a rare hematological disorder, evaluating their combined impact and the quality of the generated artificial datasets.

**Objective:**

This study aims to evaluate the privacy- and fidelity-related impact of horizontally federating SDG models in different data distribution scenarios and with different numbers of nodes, comparing them with centralized baseline SDG models.

**Methods:**

Two state-of-the-art generative models, conditional tabular generative adversarial network and FedTabDiff, were trained considering four different scenarios: (1) a nonfederated baseline with all the data available, (2) a federated scenario where the data were evenly distributed among different nodes, (3) a federated scenario where the data were unevenly and randomly distributed (imbalanced data), and (4) a federated scenario with nonindependent and identically distributed data distributions. For each of the federated scenarios, a fixed set of node quantities (3, 5, 7, 10) was considered to assess its impact, and the generated data were evaluated, attending to a fidelity-privacy trade-off.

**Results:**

The computed fidelity metrics exhibited statistically significant deteriorations (*P*<.001) up to 21% in the conditional tabular generative adversarial network and up to 62% in the FedTabDiff model due to the federation process. When comparing federated experiments trained with diverse numbers of nodes, no strong tendencies were observed, even if specific comparisons resulted in significative differences. Privacy metrics were mainly maintained while obtaining maximum improvements of 55% and maximum deteriorations of 26% between both models, although they were not statistically significant.

**Conclusions:**

Within the scope of the use case scenario in this paper, the act of horizontally federating SDG algorithms results in a loss of data fidelity compared to the nonfederated baseline while maintaining privacy levels. However, this deterioration does not significantly increase as the number of nodes used to train the models grows, even though significative differences were found in specific comparisons. The different data partition distribution configurations had no significant effect on the metrics, as similar tendencies were found for all scenarios.

## Introduction

### Overview

Acute myeloid leukemia (AML) is a group of bone marrow stem cell cancers that causes an extreme proliferation of clonal hematopoietic cells. This abnormal growth is caused by multiple cytogenetic and genetic malformations, resulting in a poorly differentiated myeloid cell accumulation in the bone marrow and the consequent spread to the blood [1].

Despite the latest scientific advances and at least 10 recently approved therapies, it is still causing 250,000 deaths yearly worldwide [2]. Moreover, even if AML accounts for about 80% of all diagnosed leukemias in adults, there are just 4.2 new cases per 100,000 people in the United States yearly, which makes it classifiable as a rare hematological disease [3]. On top of that, the proportion of AML cases among all the diagnosed leukemias worldwide increased from 18% in 1990 to 23.1% in 2017, augmenting their incidence and suggesting a potential upcoming major global public health concern [4].

According to the World Health Organization (WHO), AML can be classified into several categories: those that are derived from (1) genetic abnormalities, (2) myelodysplasia-related changes, those that are related to (3) previous chemotherapy or radiation therapies, (4) myeloid sarcomas, (5) myeloid proliferations related to Down syndrome, (6) undifferentiated and biphenotypic leukemias, and those that (7) are not otherwise specified [5,6]. In them, symptoms include bleeding, bruising, infections, fatigue, and bone pain.

The rarity of AML as a prevalent form of leukemia brings with it inherent limitations with regard to the data exploitation and consequent improvement in terms of artificial intelligence (AI) models and their application in real-world environments. First, the necessity of data is leading to the emergence of various repositories that encompass information of the disease [7], but it is important to highlight that these are often limited in size [8,9], revealing an underlying problem of data scarcity. Besides, the data protection legislation, such as the General Data Protection Regulation in Europe or the Health Insurance Portability and Accountability Act in the United States, adds a layer of complexity to the process of data sharing due to the sensitive nature of health data, as it typically consists of electronic health records (EHRs) that may contain extensive clinical or even genomic data. As a consequence, even if AML data records exist, they are unevenly distributed across different institutions, hindering any intention to make use of large amounts of data. This uneven distribution not only refers to the amount of data points available in each data silo but also to biases in them, such as racial and ethnic disparities in AML prevalence statistics [10], especially in pediatric patients [11]. This makes it even more difficult to access quality data that can be used to infer knowledge using AI.

Synthetic data (SD) is defined as artificial information that is generated from original data and a model that is trained to reproduce its characteristics and structure [12]. Thus, SD generation (SDG) is a widely used tool for creating data that mimics real-world datasets, which has been found to be helpful for augmentation tasks, as a class balancing tool, and as a privacy-enhancing technology [13]. Therefore, SD is often evaluated in terms of its fidelity with respect to the real data, its utility for downstream applications, and the privacy that it offers, the last one being one of the main topics of research in literature. In light of the current situation regarding the AML use case, SD can be considered a suitable approach for improving the current paradigm by increasing the quantity of data institution-wise. However, while SD aims to replicate real-world distributions by capturing the same range and structure as the input data, its primary focus is on addressing data scarcity rather than mitigating the problem of scattering. In this regard, SDG would be able to learn based on the local distributions and attending to the data variability found within an institution, possibly limiting the learning process and not being able to sufficiently represent a global population [14].

To mitigate the challenges related to data fragmentation and governance in distributed environments, federated learning (FL) offers a decentralized machine learning (ML) framework that enables collaborative model training across multiple institutions, ensuring that raw patient data remains local and is never transferred or shared externally. [15]. In a canonical FL environment, a model is trained in a central server using the weights each client shares after training local models on local real data [16]. Even if that local real data does not leave the node, the learned information is shared, and a global model is created, covering all the local distributions among the federated nodes and better adapting to a theoretical global distribution. That said, the type of data distributions found within a federated network directly impacts models’ behaviors, affecting communication efficiency, model convergence, and FL accuracy. Nonindependent and identically distributed (non-IID) data are currently being widely explored, as they may pose performance and privacy-related difficulties in FL contexts [17,18]. When data are distributed such that clients share the same feature space but contain distinct sets of records, the FL setting is referred to as horizontal federated learning (HFL). Conversely, when clients hold different feature subsets corresponding to the same set of records, the setting is known as vertical FL. The results presented in this work belong to HFL and may not generalize to other FL settings, like vertical FL. Hereafter, the term FL will be used analogously to HFL for the sake of clarity.

However, the adoption of technologies that combine both elements and the posterior validation of those should go hand in hand with thorough prior analyses. To do that, the contributions of this paper are:

- An evaluation of the impact of federating SDG algorithms with respect to having a model trained on all data available on the same site (centralized).
- An evaluation of the impact of the number of federated nodes on the performance of SDG models.
- An evaluation of the impact of having a randomly sampled imbalanced quantity of data in each federated node.
- An evaluation of the impact of having an imbalanced quantity of data that constitutes non-IID distributions in each federated node.

The remainder of this paper is organized as follows: the Methods section provides information about the materials used and the methodology, describing the dataset that was used, the generative model, the evaluation metrics that were implemented, and the FL setup. The next section presents the Results, while the Discussion section shows the principal results, the limitations of this work, a comparison with prior existing work, and final conclusions.

### Background

Over the last few years, the use of SD has gained momentum in several contexts. In health care, simulations and prediction research, educational and training content creation, and investigation, including the release of data, have benefited from SD use [19]. In this sense, SDG must be understood as a spectrum of possibilities regarding model selection, parameter tuning, and use case-specific contextualization. However, generating data inevitably involves sophisticated techniques, which may include generative adversarial networks (GANs), variational autoencoders (VAEs), or diffusion models, among other AI approaches.

One of the most promising uses of SD, as was mentioned previously, is the generation of artificial information that does not compromise patient privacy while maintaining its fidelity to the original counterpart. Therefore, the assessment and analyses of SD are expanding to such an extent that the expectations are surpassing those of traditional anonymization techniques, which are algorithms that typically reduce the quality of the data in terms of fidelity and utility, even attempting to combine them. In this sense, it is important to mention differential privacy (DP), which is a technique that ensures the privacy of individuals by adding random noise to the data, making it nearly impossible to determine whether any individual’s data is included in a dataset or not. DP is the most widely used privacy-enhancing technology that is being implemented in generative models to enhance data privacy, and its implementation in them depends on the intended use for the resulting SD [20-22], aligning it with the fidelity of the data. Privacy preservation of real data trades directly off with the fidelity and the utility of the generated SD, which must be maintained to ensure its suitability for downstream applications.

Regarding AI models, a GAN is composed of two networks, a generator and a discriminator, working in an adversarial manner. While the former one is supposed to minimize the loss function by generating samples that are as similar as possible to the training set, the latter is tasked with differentiating original samples from synthetic ones, trying to maximize the loss. GANs were first presented by Goodfellow et al [23] in 2014, and since then, several modifications have been proposed in order to cover a wide range of use cases. The work developed by Zhao et al [24], for example, represents the latest generation of GAN architectures, named CTAB-GAN+, which includes state-of-the-art features such as conditional generation of samples, improved loss functions, the possibility to handle both categorical and continuous data simultaneously, and DP. Narrowing down to tabular data generation for health care–related use cases, GANs were identified to be the most widely used architectures [25]. Additionally, several recent publications have addressed various unresolved questions within the field. For instance, Ramachandranpillai et al [26] introduced the bias-transforming GAN, which addresses the challenge of biased data generation in the health care domain by incorporating several information constraints inside the generation process. Moreover, various GANs are currently being experimentally tested for several use cases, as demonstrated by Akiya et al [27] in oncological clinical trials, Khan et al [28] in cardiovascular disease mortality predictions, or Dhawan and Nijhawan [29] in brain magnetic resonance imaging and chest x-ray data.

As for VAEs in the context of SDG, they are also composed of two fundamental components: the encoder and the decoder. In this context, the encoder is responsible for mapping the input training data into a latent space with a lower dimensionality, while the decoder samples new values from this latent space to reconstruct data that imitates the original inputs. Starting from the original architecture, VAEs have also undergone several modifications that help cover diverse use cases [30]. As for the latest research, Biswas and Talukdar [31] researched the enhancement of clinical documentation using both GAN and VAE-generated SD with the aim of improving patient care. Li et al [32] implemented the causal recurrent VAE, aiming to generate medical time series data. Other applications that are being investigated and include VAEs are drug dosing determinants, such as in Titar and Ramanathan [33].

Finally, diffusion models create SD by gradually transforming simple, noise-like data into complex data structures that were used during the training process. Even if this type of generative model has mostly been focused on image generation, currently, they are able to support different data types, too [34,35]. For example, Naseer et al [36] presented ScoEHR, a continuous-time diffusion model able to generate artificial EHRs. Digital pathology data was also generated with diffusion models by Pozzi et al [37].

As for generative modeling applied to rare hematological diseases, recently, D’Amico et al [38] trained a conditional tabular generative adversarial network (CTGAN) with the aim of generating myelodysplastic syndromes and AML data. Additionally, Eckardt et al [39] made their synthetic AML dataset publicly available after having considered both utility and privacy thresholds. The published synthetic dataset comprises 1606 patients generated using a CTAB-GAN+ [24]. Licandro et al [40] used a Wasserstein GAN for two distinct scenarios where differently sized datasets were used. In their research, the primary objective was to discern the embeddings of the data, enabling subsequent differentiation between blast and nonblast cells. The results show that using the generator model to learn embeddings outperforms the results obtained with baseline models, improving the area under the curve (AUC) for both dataset sizes. The study carried out by Rupapara et al [41] made use of the ADASYN [42] SD generator to balance the dataset and enhance prediction outcomes. The dataset encompassed data from various blood-related cancers, including AML. By using the ADASYN resampler, the classification models demonstrated improved accuracy.

Regarding FL-related studies, several experiments have been conducted to overcome the data scattering issue. For example, the study carried out by Linardos et al [43] simulates a federated environment consisting of four nodes. The study aimed to help diagnose hypertrophic cardiomyopathy diseases, the results supporting the effectiveness of FL by achieving better AUC results than with a collaborative data-sharing framework. The work presented by Liu et al [44] focused on using FL to achieve a deep learning model that makes use of EHRs to predict patient mortality, which they called FADL. The work presented by Azizi et al [45] also used EHR information scattered among 50 nodes, each of which contained 560 patients, to predict mortality. However, in this case, they used a clustering method and used community-based FL, surpassing the performance of the canonical FL environment across various scenarios.

Both techniques, SDG and FL, have demonstrated their effectiveness in various sectors and use cases. The combination of both is being investigated to such an extent that inherently federated generative models are being published in the literature, such as the private FL-GAN model [46]. Focusing on health care–related topics, in Azizi et al [45], a framework for cardiovascular data based on an FL architecture of two nodes and a generative model using sequential trees is shown. The study presented by Behera et al [47] demonstrates the implementation of a GAN within a federated environment, called FedSyn. In addition to applying DP, thereby enhancing data protection, the researchers used the CIFAR10 and Modified National Institute of Standards and Technology datasets for their analyses. The research outlined by Xin et al [48] uses a federated GAN augmented with DP, trained on both Modified National Institute of Standards and Technology and CelebA datasets. The authors analyzed the privacy of the generated data offered against the original one, concluding an improved privacy against membership inference attacks (MIA). However, despite the combination of both SDG and FL explored in different studies, many aspects of this mixture still require evaluation.

## Methods

### AML Dataset

The AML dataset used to perform the research of this paper was accessed from the work developed by Tazi et al [49] and its associated GitHub repository [50].

Among the available datasets in the repository, the paper_full_data_validation dataset was chosen for this research. All the genetic mutation-related variables were discarded, preserving clinical, demographic, and disease-related information. Multimedia Appendix 1 includes a description of the variables used in the experiments. The variable selection was carried out to maintain acceptable sample-to-feature ratios across various federated configurations regarding node quantities, as the original dataset comprised 130 features. Having a low number of samples and too many variables would have limited the experiments in this regard, as highly overfitted models would appear. The resulting dataset consisted of 1540 samples and 12 features, from which the categorical ones were label encoded in the preprocessing stage. All these preprocessing steps were applied before the data were split into different client nodes for the sake of simplicity. Applying these specific preprocessing steps to our federated experiments would entail just an additional preliminary step in the training process, which was omitted by manually preprocessing the data.

### Ethical Considerations

That study was conducted following the completion of informed consent forms by all the included participants. In addition, all the relevant ethical guidelines were followed, and necessary Institutional Review Board and ethics committee approvals were obtained. The trial was conducted in accordance with the tenets of the Helsinki Declaration, and it was sponsored by Cardiff University and approved by the Wales research ethics committee (protocol 08/MRE09/29). The analysis of the data in the original study was approved by the Memorial Sloan Kettering Cancer Center Institutional Review Board (protocol x20-064). All the raw data were deposited in the European Genome Phenome Archive (reference EGAS00001000570). In this paper, all the information that allows the identification of any of the participants was omitted in accordance with privacy and confidentiality standards. The authors in Tazi et al [49] and Tazi [50], who performed the original analysis on the data, do not bear any responsibility for the further research reported in this work.

### Generative Models

The first generative model that was selected for this experiment is the CTGAN [51], as it was recently reported to have one of the most appropriate generators among different GAN and VAE architectures [52]. Additionally, it is implemented in a way that models the relationships between imbalanced variable distributions [53]. The Synthetic Data Vault [54] CTGAN implementation was used in this work, even if some modifications had to be implemented for the correct use of the model in a federated environment.

Regarding the model parameters used on the CTGAN, the default parameters presented in the Synthetic Data Vault (v0.18.0) implementation were used. The same architecture was set for both the discriminator and the generator with a two-layer hidden structure, both containing 256 units each. The learning rates of both objects were set to 2×10^–4^, with the decay fixed to 1×10^–6^. A batch size of 500 samples was defined along with an embedding dimension of 128 samples. The discriminator was updated along with the generator at every training step, and a 10-sample group (pac parameter) was introduced into the discriminator each time it was applied.

On the other hand, a diffusion model was also included. Diffusion models [55] are a newer class of generative models that have demonstrated superior performance compared to other tabular generative models [56,57]. The implementation used in this work is the one presented by Sattarov et al [58], where the authors claim high fidelity and privacy metrics were obtained in their experiments.

As for the FedTabDiff model parameters, default values were also chosen. Specifically, the number of diffusion steps was set to 500, and the multilayer perceptron layers were configured as {512,512}. To ensure comparability across models, a consistent batch size of 500 was used, and the learning rate for the deep learning models was fixed to 1×10^–4^, with a linear scheduler. Additionally, the diffusion beta start was set to 1×10^–4^, and the beta end was set to 0.02. It is worth mentioning that the model was slightly modified in order for it to interact multiple times with similar data, with the idea of the results being comparable among experiments.

As mentioned previously, in order for each participant node to transform the data in the same manner and to avoid averaging mismatches, one-hot encoding for discrete columns and Gaussian mixture transformations for continuous variables were fit using the whole dataset, also being able to avoid unseen classes in federated nodes. The objects were then included in each client with the aim of transforming each data partition in situ and using the same mapping.

Regarding the number of epochs to be performed during the training process of the models, different experiments were empirically conducted on the baseline model with 500, 1000, 1500, 2000, and 3000 epochs. The optimal configuration was proven to be 500 epochs, as increasing the iterations did not show any significant improvement in the generated synthetic sample quality. All the federated models were trained for 500 epochs for the experiments to be comparable. In addition, the number of federation rounds was set to 500.

### Evaluation Metrics

The generated SD was analyzed to gauge its fidelity and privacy with respect to the real data. In the scope of this work, fidelity is defined as the degree to which the generated SD replicates the characteristics, patterns, correlations, and distributions of the real data. While a high fidelity means the SD resembles the real data well, a low fidelity would indicate poor learning by the model generators. On the other hand, privacy is defined as the extent to which the generated data protects sensitive information from being disclosed in the original dataset. In this section, the methods and metrics to evaluate the SD are described.

Considering a simulated FL scenario, the comparison was performed against the whole real data, thus being able to compare the performance of each setup against the nonfederated scenario as a baseline. Inspired by usual ML cross-validation, 10 different synthetic datasets were generated with each model, allowing a separate evaluation for each of them. The results were then averaged to provide a more robust perspective on their generalizability. The set of metrics calculated in each fold also enabled the execution of statistical tests for significance.

In order to assess intervariable correlations, the *ϕ*_k_ coefficient [59] and the Vendi Score (VS) [60] metrics were implemented. On the one hand, the *ϕ*_k_ coefficient is based on the refinement of Pearson hypothesis tests. However, unlike Pearson hypothesis, *ϕ*_k_ can calculate correlations with both numerical and categorical variables, the higher the values, suggesting better intervariable relations. Moreover, *ϕ*_k_ can capture nonlinear relationships. Correlation matrices were generated for both real and synthetic versions of the datasets using the *ϕ*_k_ coefficient, and the cosine similarity (CS) metric was used to obtain a quantitative measure that compares them, which is defined as 1–*d*_cos_:



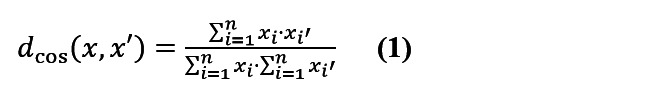



Where *x*_i_ is a real sample and *x_i_*' is a synthetic counterpart. A low CS metric suggests the two matrices do not look alike, while higher values imply higher similarities between them.

On the other hand, the VS is a novel metric that computes the diversity of a given dataset without the need to compare it against another set of data. This score requires defining a positive semidefinite similarity function, which was set to be the CS in this case [61]. Accordingly, the VS of just the numerical attributes was computed due to the CS only being applicable to numerical features. In the following equation, the mathematical expression for the VS can be observed.



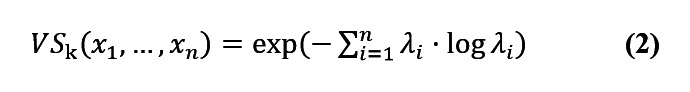



Where *k* is a given similarity function and *λ*_n_ are the eigenvalues of *K/n*, and *K* is the kernel matrix.

Furthermore, a data labeling analysis (DLA) was performed. In this procedure, an ML classifier is trained to ascertain its ability to differentiate between synthetic and real samples, mimicking the functionality of a GAN discriminator. Due to the characteristics of the analysis, the classification process was evaluated using the *F*_1_-score metric since it is sensitive to the class distributions, making it a reliable metric when labels are imbalanced, and the recall score, as it returns the number of correctly identified synthetic samples. On top of that, the AUC curve was calculated. Regarding the trained ML models for the DLA, the LazyPredict classifier [62] object was used to train various models per iteration. The best classifier was chosen for each fold to account for the most restrictive case, while the mean and standard deviation were calculated in the process.

On top of those, the Hellinger distance was chosen to quantify the similarity between two probability distributions, offering a bounded metric that is interpretable and less sensitive to outliers than other distance calculations, such as the Wasserstein one [63]. Finally, the depth versus depth plot (DD-plot) was used, which is a nonparametric method that can evaluate the multivariate distributional similarity between two distributions (real and synthetic, in this case). While the DD-plot aims to represent the depth of a real distribution with respect to the associated synthetic depth in a graphical way, the coefficient of determination (*R*^2^) is proposed in the literature to obtain an analytical value from it. Both the real and SD depths are more similar when *R*^2^ is higher, meaning that the DD-plot better fits a theoretical optimal function *x*=*y*.

With respect to privacy metrics, four types of attacks were conducted: MIAs, attribute inference attacks (AIAs), linkability attacks, and singling out attacks. MIA and AIA assessments were conducted using the Anonymeter tool [64]. In MIAs, an adversary is simulated to assess whether a specific data point was part of the training dataset used to train a generative model, thereby posing potential privacy risks. The attack methodology involves computing distance measures between pairs of records and applying a threshold to distinguish between high-risk matches and those considered safe. In the context of this experiment and following the work of Hernandez et al [63], a Gower distance of 0.05 was defined, which is a similarity measure that may be used to handle multitype data within the same dataset.

In contrast, AIAs occur when an adversary attempts to infer sensitive information that was not originally disclosed with the dataset. AIAs seek to extract additional private information about individuals, even if their membership is already known or assumed. In this case, risks are calculated variable-wise as each one may pose differently ranked sensitivities. For numerical variables, an AIA is considered successful if the predicted value falls within a predefined confidence interval of the true value, which is defined to 0.05 in the scope of this work. For categorical variables, a correct prediction requires an exact match. This evaluation is conducted across all variables individually, and the average success rate is used to compute the overall attribute inference risk.

Regarding linkability attacks, their goal is to associate attributes from two or more records with the same individual or group, either using a single dataset or multiple ones. If the known attributes and a synthetic dataset allow the linkage to the real dataset, revealing sensitive information, the attack is considered successful. Singling out attacks, on the other hand, occurs when unique data records can be identified based on a distinct combination of attributes within the real data. In the scope of this work, the most stringent approach was adopted, using the conjunction of all attributes to perform the attacks (multivariate singling out).

### Experimental Setup

**Figure 1:**
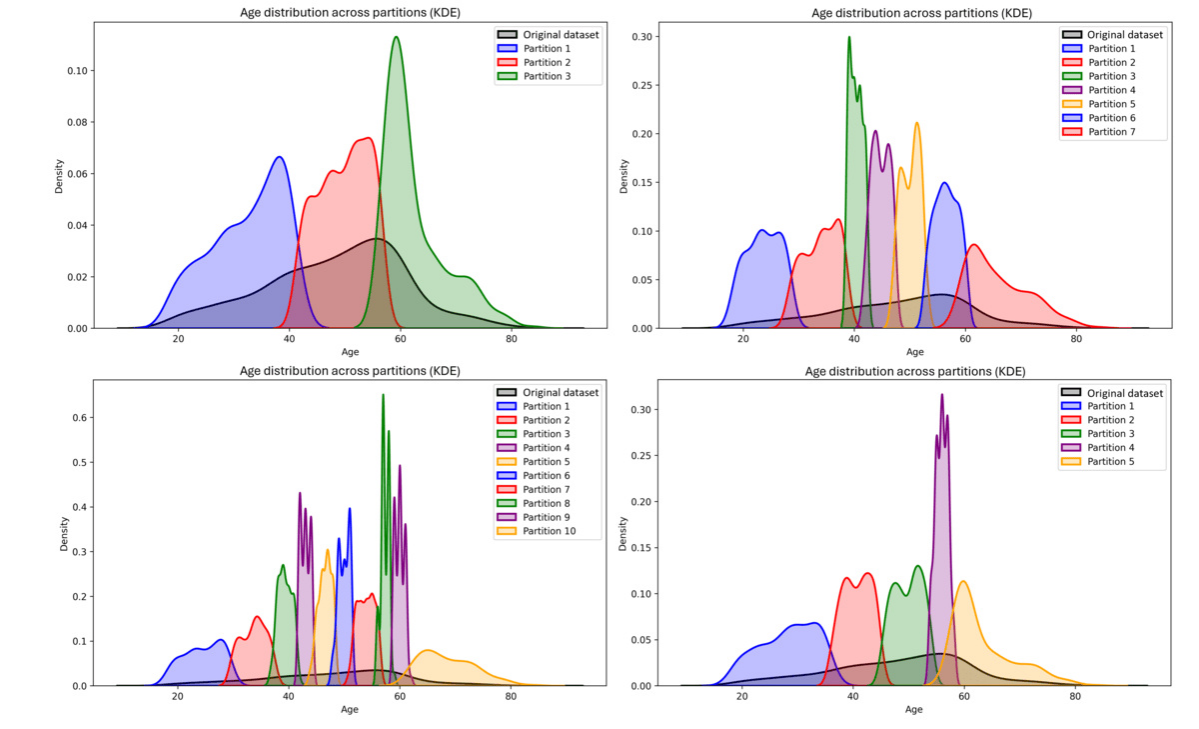
Generated non-IID age-dependent distribution plots. The top left shows the scenario with 3 nodes, the top right shows the 7-node scenario, the bottom left shows the 10-node scenario, and the bottom right shows the 5-node one. KDE: kernel density estimate; non-IID: nonindependent and identically distributed.

In this experiment, a comparison between nonfederated and federated generative models was performed for three different federated scenarios. In the first one, the data quantity was assumed to be evenly distributed across the participant nodes (from now on, B scenario, for balanced), while for the second, the data points were randomly split, creating partitions with uneven sample quantities (from now on, IB scenario). In the third federated scenario, non-IID distributions (Figure 1) were built depending on the age variable (from now on, IB_non-iid_ scenario).

For the three scenarios, the data were partitioned prior to the model training phase, allowing for traceability and higher result reproducibility. The partitions for the B scenario were created by randomly selecting n/N samples for each of the nodes, being the total number of samples in the original dataset and being the number of nodes that participate in a specific federated experiment. On the other hand, the IB scenario partitions were created so that for a specific N, N–1 nodes were trained on 5% of the data that was chosen randomly, and the th node was trained on the remaining samples. The IB_non-iid_ scenario was created by sampling age-dependent data points from Dirichlet (α=10) distributions [65]. The distribution of categorical variable labels across the partitions, as well as the exact number of samples per scenario, can be consulted in Multimedia Appendix 2, showing bias among scenarios. In this analysis, the federation was evaluated for a set of N∈{3,5,7,10}.

Regarding the aggregation method selection, a preliminary study was performed using the federated average (FedAvg), the adaptive federation optimization (FedOpt), and the federated optimization for heterogeneous networks (FedProx) algorithms. The most challenging experiment scenarios (10 nodes and non-IID scenarios) were tested with the three algorithms, evaluating the models using the metrics that were presented in the previous section. The results either demonstrated that FedAvg was the best-performing algorithm or showed no statistically significant improvement when comparing to FedOpt or FedProx algorithms. Therefore, the experiments intended to evaluate the contributions defined in the introduction section of this paper were trained by averaging the model weights coming from each node and attending to the number of samples each one contained (FedAvg). The results of the preliminary analysis can be found in Multimedia Appendix 3.

**Figure 2:**
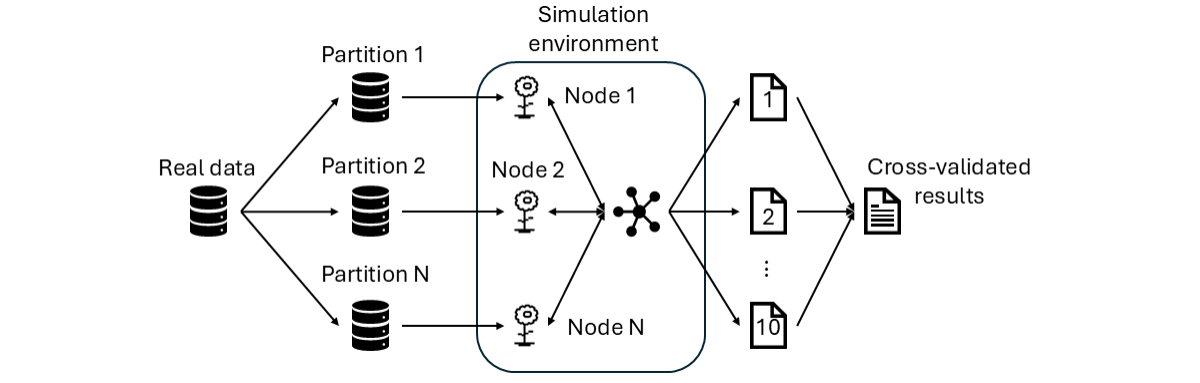
Experiment execution framework, including data partitioning, model training, and synthetic data evaluation processes.

The Flower 1.7.0 Framework [66] was used in order to federate the models using the simulation module. The experiments were performed in a high-performance computing cluster, and they were allocated for 10 central processing unit and 2 graphics processing unit jobs, having initiated a Ray actor cluster prior to executing federation rounds. Figure 2 represents the workflow that was carried out during the experiment execution, where data partition, model training, and evaluation phases are shown.

## Results

### Overview

The results of the study are presented in this section, comparing the three presented federated scenarios with the baseline model, both for the CTGAN and the FedTabDiff. All statistical tests were performed for a significance level of .05 using the averaged results, while some variable-specific metrics can be checked in Multimedia Appendices 4 and 5.

### CTGAN

Starting with the baseline nonfederated CTGAN model fidelity evaluation, the coefficient results showed a mean CS of 0.930 (standard deviation 0.002). Regarding the DLA execution, the obtained AUC was 0.796 (standard deviation 0.026), the *F*_1_-score was 0.872 (standard deviation 0.015), and the recall metric was 0.958 (standard deviation 0.009). The VS resulted in a mean value of 1.405 (standard deviation 0.004) in the SD, compared to the VS obtained in the real dataset of 1.406. To finish, the average Hellinger distance was 0.223 (standard deviation 0.002). Regarding the privacy evaluation, the MIA demonstrated no significant membership inference risk, while the averaged AIA resulted in a 5% risk for attribute information to be inferred.

Regarding the federated models that were trained with balanced datasets (the B scenario), most of the performed experiments showed statistically significant differences in fidelity metrics with respect to the baseline scenario (*P*<.001), even with variations in the number of nodes (Table 1). Statistical significance was found in singling out attack metrics, while the rest of the privacy measures did not significantly vary from the baseline.

**Table 1:** Fidelity and privacy metric results of the CTGAN^a^ for the B scenario^b^.

Metric				Baseline		3N	5N		7N		10N
**Fidelity**											
	**CS^c^*ϕ*_k_**										
		µ	0.930		0.842		0.846	0.845		0.849	
		σ	0.002		0.005		0.003	0.003		0.003	
		*t* test (*df*)	—^d^		45.525 (18)		67.770 (18)	74.556 (18)		78.915 (18)	
		*P* value	—		<.001		<.001	<.001		<.001	
	**DLA^e^ AUC ^f^**										
		µ	0.796		0.965		0.962	0.946		0.965	
		σ	0.026		0.008		0.008	0.008		0.009	
		*t* test (*df*)	—		19.818 (18)		19.333 (18)	17.391 (18)		19.402 (18)	
		*P* value	—		<.001		<.001	<.001		<.001	
	**DLA *F*_1_-score**										
		µ	0.872		0.965		0.961	0.945		0.964	
		σ	0.015		0.007		0.008	0.008		0.009	
		*t* test (*df*)	—		17.940 (18)		16.931 (18)	13.853 (18)		16.935 (18)	
		*P* value	—		<.001		<.001	<.001		<.001	
	**DLA recall**										
		µ	0.958		0.969		0.948	0.940		0.968	
		σ	0.009		0.012		0.009	0.012		0.010	
		*t* test (*df*)	—		2.402 (18)		2.644 (18)	3.791 (18)		2.316 (18)	
		*P* value	—		.02		.02	.001		.03	
	**VS^g^**										
		µ	1.405		1.437		1.442	1.337		1.251	
		σ	0.004		0.002		0.001	0.004		0.006	
		*t* test (*df*)	—		24.969 (18)		31.451 (18)	39.839 (18)		66.590 (18)	
		*P* value	—		<.001		<.001	<.001		<.001	
	
		µ	0.223		0.229		0.223	0.220		0.221	
		σ	0.002		0.010		0.002	0.002		0.002	
		*t* test (*df*)	—		1.767 (18)		0.165 (18)	2.930 (18)		2.517 (18)	
		*P* value	—		.09		.87	.009		.02	
	**Depth versus depth-plot *R*^2^**										
		µ	0.948		0.950		0.668	0.828		0.619	
		σ	0.011		0.007		0.006	0.007		0.005	
		*t* test (*df*)	—		0.525 (18)		71.176 (18)	29.506 (18)		86.088 (18)	
		*P* value	—		.61		<.001	<.001		<.001	
**Privacy**											
	**MIA^h^**										
		µ	0		0^i^		0^i^	0^i^		0^i^	
		σ	0		0		0	0		0	
		*t* test (*df*)	—		—		—	—		—	
		*P* value	—		—		—	—		—	
	**AIA^j^**										
		µ	0.045		0.038		0.055	0.038		0.039	
		σ	0.025		0.018		0.031	0.019		0.009	
		*t* test (*df*)	—		1.496 (18)		1.000 (18)	1.414 (18)		1.667 (18)	
		*P* value	—		.15		.33	.17		.11	
	**Linkability**										
		µ	0.001		0.001^k^		0.001^k^	0.001^k^		0^i^	
		σ	0.003		0.003		0.003	0.003		0	
		*t* test (*df*)	—		—		—	—		—	
		*P* value	—		—		—	—		—	
	**Singling out**										
		µ	0.123		0.048		0.018	0.048		0.046	
		σ	0.045		0.016		0.018	0.025		0.025	
		*t* test (*df*)	—		4.954 (18)		6.785 (18)	4.548 (18)		4.645 (18)	
		*P* value	—		<.001		<.001	<.001		<.001	

^a^CTGAN: conditional tabular generative adversarial network.

^b^*t* tests were performed between the baseline and each federated experiment. Significance level is .05 for all statistical tests.

^c^CS: cosine similarity.

^d^Not applicable.

^e^DLA: data labeling analysis.

^f^AUC: area under the curve.

^g^VS: Vendi Score.

^h^MIA: membership inference attack.

^i^*t* tests were not performed for these due to the standard deviation being zero.

^j^AIA: attribute inference attack.

^k^*t* tests were not performed for these due to the results being the same as the ones found in the baseline metrics.

Specifically, intervariable correlations were shown to be more distorted than the ones presented by the baseline model, and the DLA suggested that the synthetic samples that were generated by federated models are prone to being detected more easily than the ones generated by the baseline model, although variable-wise metrics such as the Hellinger distance did not demonstrate too different results.

In the IB scenario, most of the performed experiments showed high statistical significances (*P*<.001) with respect to the baseline, too (Table 2). Intervariable correlations, DLA metrics, and VS values were shown to be quite different from the baseline model, attending the statistical tests, while the Hellinger distances did not show too big a difference. In this case, the 10N experiment showed statistically significant differences with the AIA metric obtained in the baseline, suggesting an improvement in privacy while deteriorating the fidelity values. All the experiments in this scenario supported the privacy improvement suggestion, as all the multivariate singling out attacks proved to perform better in federated setups. However, no specific tendency can be observed while increasing the number of nodes in this sense.

The IB_non-iid_ scenario followed the same overall patterns found in the previous two scenarios, showing statistically significant differences in fidelity metrics but with no difference in the performed privacy metrics other than those found for multivariate singling out attacks (Table 3).

**Table 2:** Fidelity and privacy metric results of the CTGAN^a^ for the IB scenario^b^.

Metric				Baseline		3N	5N		7N		10N
**Fidelity**											
	**CS ^c^ *ϕ*_k_**										
		µ	0.930		0.848		0.841	0.839		0.847	
		σ	0.002		0.002		0.003	0.005		0.001	
		*t* test (*df*)	—^d^		87.050		84.898	56.581		110.235	
		*P* value	—		<.001		<.001	<.001		<.001	
	**DLA^e^ AUC^f^**										
		µ	0.796		0.983		0.993	0.968		0.938	
		σ	0.026		0.004		0.004	0.005		0.009	
		*t* test (*df*)	—		23.187 (18)		23.700 (18)	20.550 (18)		16.342 (18)	
		*P* value	—		<.001		<.001	<.001		<.001	
	**DLA *F*_1_-score**										
		µ	0.872		0.988		0.993	0.968		0.938	
		σ	0.015		0.004		0.004	0.005		0.008	
		*t* test (*df*)	—		24.086 (18)		25.109 (18)	19.405 (18)		12.136 (18)	
		*P* value	—		<.001		<.001	<.001		<.001	
	**DLA recall**										
		µ	0.958		0.979		0.986	0.964		0.928	
		σ	0.009		0.007		0.008	0.008		0.014	
		*t* test (*df*)	—		5.749 (18)		7.472 (18)	1.581 (18)		5.666 (18)	
		*P* value	—		<.001		<.001	.13		<.001	
	**VS^g^**										
		µ	1.405		1.360		1.424	1.436		1.360	
		σ	0.004		0.011		0.003	0.003		0.011	
		*t* test (*df*)	—		27.714 (18)		11.833 (18)	21.772 (18)		12.155 (18)	
		*P* value	—		<.001		<.001	<.001		<.001	
		µ	0.223		0.222		0.216	0.223		0.217	
		σ	0.002		0.001		0.002	0.002		0.002	
		*t* test (*df*)			0.976 (18)		7.892 (18)	0.119 (18)		5.931 (18)	
		*P* value			.34		<.001	.90		<.001	
	**Depth versus depth-plot *R* ^2^**										
		µ	0.948		0^h^		0^h^	0.737		0^h^	
		σ	0.011		0		0	0		0	
		*t* test (*df*)	—		—		—	56.069 (18)		—	
		*P* value	—		—		—	<.001		—	
**Privacy**											
	**MIA^i^**										
		µ	0		0^h^		0^h^	0^h^		0^h^	
		σ	0		0		0	0		0	
		*t* test (*df*)	—		—		—	—		—	
		*P* value	—		—		—	—		—	
	**AIA^j^**										
		µ	0.045		0.052		0.061	0.041		0.029	
		σ	0.025		0.019		0.029	0.016		0.017	
		*t* test (*df*)	—		0.076 (18)		0.768 (18)	1.400 (18)		2.437 (18)	
		*P* value	—		.94		.45	.27		.03	
	**Linkability**										
		µ	0.001		0.001^k^		0^h^	0.002		0.002	
		σ	0.003		0.003		0	0.004		0.004	
		*t* test (*df*)	—		—		—	0.600 (18)		0.600 (18)	
		*P* value	—		—		—	.56		.56	
	**Singling out**										
		µ	0.123		0.024		0.025	0.030		0.060	
		σ	0.045		0.022		0.015	0.014		0.025	
		*t* test (*df*)	—		6.181 (18)		6.457 (18)	6.189 (18)		3.798 (18)	
		*P* value	—		<.001		<.001	<.001		.001	

^a^CTGAN: conditional tabular generative adversarial network.

^b^*t* tests were performed between the baseline and each federated experiment. Significance level is .05 for all statistical tests.

^c^CS: cosine similarity.

^d^Not applicable.

^e^DLA: data labeling analysis.

^f^AUC: area under the curve.

^g^VS: Vendi Score.

^h^*t* tests were not performed for these due to the standard deviation being zero.

^i^MIA: membership inference attack.

^j^AIA: attribute inference attack.

^k^*t* tests were not performed for these due to the results being the same as the ones found in the baseline metrics.

**Table 3:** Fidelity and privacy metric results of the CTGAN^a^ for the IBnon-iid scenario^b^.

Metric				Baseline	3N		5N		7N		10N
**Fidelity**											
	CS^c^ *ϕ*_k_										
		µ	0.930		0.848	0.842		0.841		0.842	
		σ	0.002		0.003	0.001		0.004		0.003	
		*t* test (*df*)	—^d^		82.533 (18)	111.330 (18)		69.045 (18)		83.862 (18)	
		*P* value	—		<.001	<.001		<.001		<.001	
	**DLA^e^ AUC^f^**										
		µ	0.796		0.977	0.948		0.983		0.950	
		σ	0.026		0.006	0.010		0.005		0.006	
		*t* test (*df*)	—		21.572 (18)	17.233 (18)		22.408 (18)		18.339 (18)	
		*P* value	—		<.001	<.001		<.001		<.001	
	**DLA *F*_1_-score**										
		µ	0.872		0.976	0.947		0.982		0.950	
		σ	0.015		0.005	0.010		0.005		0.006	
		*t* test (*df*)	—		21.033 (18)	13.353 (18)		22.724 (18)		15.602 (18)	
		*P* value	—		<.001	<.001		<.001		<.001	
	**DLA recall**										
		µ	0.958		0.960	0.938		0.985		0.952	
		σ	0.009		0.010	0.019		0.009		0.013	
		*t* test (*df*)	—		0.383 (18)	3.039 (18)		6.891 (18)		1.195 (18)	
		*P* value	—		.71	.007		<.001		.25	
	**VS^g^**										
		µ	1.405		1.440	1.445		1.198		1.367	
		σ	0.004		0.001	0.001		0.010		0.004	
		*t* test (*df*)	—		28.834 (18)	33.888 (18)		62.753 (18)		21.978 (18)	
		*P* value	—		<.001	<.001		<.001		<.001	
		µ	0.223		0.214	0.214		0.215		0.213	
		σ	0.002		0.002	0.003		0.002		0.002	
		*t* test (*df*)	—		8.801 (18)	8.147 (18)		8.333 (18)		11.928 (18)	
		*P* value	—		<.001	<.001		<.001		<.001	
	**Depth versus depth-plot *R*^2^**										
		µ	0.948		0.708	0.809		0^h^		0.847	
		σ	0.011		0.021	0.006		0		0.004	
		*t* test (*df*)	—		32.423 (18)	35.585 (18)		—		27.404 (18)	
		*P* value	—		<.001	<.001		—		<.001	
**Privacy**											
	**MIA^i^**										
		µ	0		0^h^	0^h^		0^h^		0^h^	
		σ	0		0	0		0		0	
		*t* test (*df*)	—		—	—		—		—	
		*P* value	—		—	—		—		—	
	**AIA^j^**										
		µ	0.045		0.048	0.044		0.049		0.057	
		σ	0.025		0.022	0.023		0.024		0.032	
		*t* test (*df*)	—		0.366 (18)	0.799 (18)		0.325 (18)		0.362 (18)	
		*P* value	—		.72	.44		.75		.72	
	**Linkability**										
		µ	0.001		0.002	0.001^k^		0.001^k^		0^h^	
		σ	0.003		0.006	0.003		0.003		0	
		*t* test (*df*)	—		0.447 (18)	—		—		—	
		*P* value	—		.66	—		—		—	
	**Singling out**										
		µ	0.123		0.030	0.029		0.033		0.021	
		σ	0.045		0.020	0.021		0.019		0.021	
		*t* test (*df*)	—		5.877 (18)	5.900 (18)		5.768 (18)		6.136 (18)	
		*P* value	—		<.001	<.001		<.001		<.001	

^a^CTGAN: conditional tabular generative adversarial network.

^b^*t* tests were performed between the baseline and each federated experiment. Significance level is .05 for all statistical tests.

^c^CS: cosine similarity.

^d^Not applicable.

^e^DLA: data labeling analysis.

^f^AUC: area under the curve.

^g^VS: Vendi Score.

^h^*t* tests were not performed for these due to the standard deviation being zero.

^i^MIA: membership inference attack.

^j^AIA: attribute inference attack.

^k^*t* tests were not performed for these due to the results being the same as the ones found in the baseline metrics.

**Figure 3:**
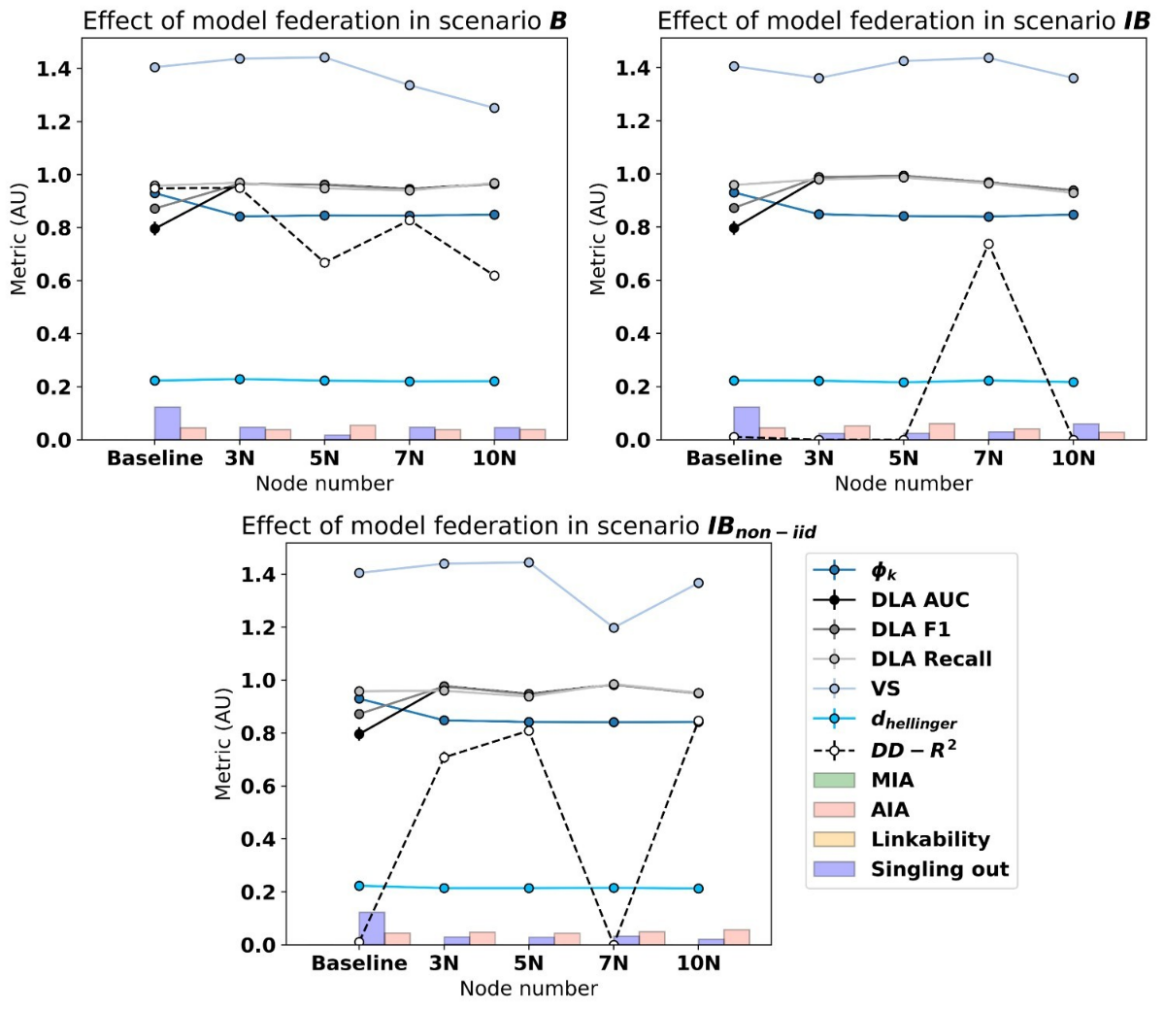
Graphical comparison of federated scenarios regarding fidelity and privacy metrics using the CTGAN model. AIA: attribute inference attack; AUC: area under the curve; CTGAN: conditional tabular generative adversarial network; DD: depth versus depth; DLA: data labeling analysis; MIA: membership inference attack; non-IID: nonindependent and identically distributed; VS: Vendi Score.

Gathering all the results in a single figure, similar tendencies can be observed in the three scenarios, where lower correlation values and higher DLA-related metrics can be found among the federated models with respect to the baseline model. The VS metric fluctuated most among the scenarios along with the DD-plot *R*^2^, offering insight regarding the variability of each generated SD. Hellinger distances were found not to fluctuate even when comparing centralized and federated models, while a difference in privacy protection measures is observable in singling out attacks (Figure 3).

Now, considering federated experiment pairs (ie, comparing 3N experiments with 5N, 5N with 7N, and 7N with 10N) to evaluate if additional federated nodes impact the SD quality in terms of fidelity and privacy, no clear tendencies can be observed in either scenario (Table 4). Privacy metrics did not show statistical significance; therefore, assuming no improvement was achieved in terms of privacy, even though the baseline centralized model offered good results already. Among the fidelity metrics, the CS of the was found to differ between pairs of experiments in some cases, but no specific trend was detected. The same occurred for the DLA-related metrics, where some pairs pointed out significant differences. All the VS metrics were found to be different, even if no improvement or deterioration trend was found, and the Hellinger distance metric varied depending on the scenario, just as the DD-plot *R*^2^.

**Table 4:** *t* test results for CTGAN^a^ experiment pairs^b^.

Metric				3N-5N				5N-7N				7N-10N		
				*t* test (*df*)		*P* value		*t* test (*df*)		*P* value		*t* test (*df*)		*P* value
**B scenario**														
	**Fidelity**													
		CS^c^ *ϕ*_k_	1.858 (18)		.08		0.522 (18)		.61		2.939 (18)		.008	
		DLA^d^ AUC^e^	0.895 (18)		.38		4.522 (18)		<.001		4.944 (18)		<.001	
		DLA *F*_1_-score	0.875 (18)		.39		4.495 (18)		<.001		4.951 (18)		<.001	
		DLA Recall	4.612 (18)		<.001		1.621 (18)		.12		5.553 (18)		<.001	
		VS^f^	7.949 (18)		<.001		82.267 (18)		<.001		36.200 (18)		<.001	
		*d*_hellinger_	1.704 (18)		.11		2.815 (18)		.01		0.714 (18)		.48	
		DD-plot^g^ *R*^2^	98.714 (18)		<.001		55.822 (18)		<.001		77.093 (18)		<.001	
	**Privacy**													
		MIA^h,i^	—^j^		—		—		—		—		—	
		AIA^k^	1.000 (18)		.33		1.000 (18)		.33		0.045 (18)		.96	
		Linkability^i,l^	—		—		—		—		—		—	
		Singling out	3.702 (18)		.002		2.898 (18)		.01		0.120 (18)		.91	
**IB scenario**														
	**Fidelity**													
		CS *ϕ*_k_	6.296 (18)		<.001		1.447 (18)		.16		5.676 (18)		<.001	
		DLA AUC	2.165 (18)		.04		11.306 (18)		<.001		9.083 (18)		<.001	
		DLA *F*_1_-score	2.192 (18)		.04		11.485 (18)		<.001		9.026 (18)		<.001	
		DLA Recall	2.088 (18)		.051		6.129 (18)		<.001		6.967 (18)		<.001	
		VS	14.629 (18)		<.001		9.680 (18)		<.001		21.062 (18)		<.001	
		*d*_hellinger_	8.239 (18)		<.001		7.517 (18)		<.001		5.708 (18)		<.001	
		DD-plot *R*^2i^	—		—		—		—		—		—	
	**Privacy**													
		MIA^i^	—		—		—		—		—		—	
		AIA	0.743 (18)		.47		1.796 (18)		.09		1.592 (18)		.13	
		Linkability^i,l^	—		—		—		—		—		—	
		Singling out	0.192 (18)		.85		0.719 (18)		.48		3.074 (18)		.007	
**IB_non-iid_ scenario**														
	**Fidelity**													
		CS *ϕ*_k_	7.211 (18)		<.001		0.985 (18)		.34		0.476 (18)		.64	
		DLA AUC	7.895 (18)		<.001		9.805 (18)		<.001		14.043 (18)		<.001	
		DLA *F*_1_-score	7.705 (18)		<.001		9.718 (18)		<.001		14.039 (18)		<.001	
		DLA Recall	3.192 (18)		.005		7.086 (18)		<.001		6.784 (18)		<.001	
		VS	15.145 (18)		<.001		79.749 (18)		<.001		50.058 (18)		<.001	
		*d*_hellinger_	0.120 (18)		.91		0.585 (18)		.57		2.085 (18)		.054	
		DD-plot *R*^2i^	14.916 (18)		<.001		—		—		—		—	
	**Privacy**													
		MIA^i^	—		—		—		—		—		—	
		AIA	0.435 (18)		.67		0.441 (18)		.66		0.615 (18)		.54	
		Linkability^i,l^	0.447 (18)		.66		—		—		—		—	
		Singling out	0.131 (18)		.90		0.457 (18)		.65		1.577 (18)		.13	

^a^CTGAN: conditional tabular generative adversarial network.

^b^Significance level is .05 for all statistical tests.

^c^CS: cosine similarity.

^d^DLA: data labeling analysis.

^e^AUC: area under the curve.

^f^VS: Vendi Score.

^g^DD-plot: depth versus depth plot.

^h^MIA: membership inference attack.

^i^*t* tests were not performed for these due to the standard deviation being zero.

^j^Not applicable.

^k^AIA: attribute inference attack.

^l^*t* tests were not performed for these due to the results being the same as the ones found in the baseline metrics.

### FedTabDiff

Regarding the baseline FedTabDiff model, a CS *ϕ*_k_ value of 0.866 was obtained, while results above 0.990 were obtained for all DLA-related metrics. The VS was 1.417, while the Hellinger distance was 0.339, and the DD-plot *R*^2^ was 0.971. The privacy analyses demonstrated there was no membership inference risk, while the linkability risk was minimal. Values of 0.031 for the AIA and 0.156 for the singling out were obtained.

The FedTabDiff models that were trained with balanced datasets (the B scenario) showed statistical significance in most of the fidelity metrics when compared to the baseline scenario (*P*<.001), while the tests carried out with the privacy metrics were found to be nonsignificant. Therefore, fidelity metrics worsened while the number of nodes augmented, but privacy seemed not to be affected (Table 5).

**Table 5:** Fidelity and privacy metric results of FedTabDiff for the B scenario^a^.

Metric				Baseline		3N	5N		7N		10N
**Fidelity**											
	**CS^b^ *ϕ*_k _**										
		µ	0.866		0.854		0.674	0.873		0.645	
		σ	0.010		0.011		0.185	0.008		0.168	
		*t* test (*df*)	—^c^		2.489 (18)		3.121 (18)	1.588 (18)		3.931 (18)	
		*P* value	—		.02		.006	.13		<.001	
	**DLA^d^ AUC^e^**										
		µ	0.992		0.981		0.989	0.994		0.997	
		σ	0.001		0.001		0.001	0.001		0.003	
		*t* test (*df*)	—		19.152 (18)		5.594 (18)	5.965 (18)		14.631 (18)	
		*P* value	—		<.001		<.001	<.001		<.001	
	**DLA *F*_1_-score**										
		µ	0.997		0.994		0.999	1		0.999	
		σ	0.003		0.002		0.001	0		0.001	
		*t* test (*df*)	—		2.642 (18)		1.886 (18)	2.899 (18)		2.612 (18)	
		*P* value	—		.02		.08	.009		.02	
	**DLA recall**										
		µ	0.999		0.997		0.999	1		1	
		σ	0.002		0.003		0.002	0		0	
		*t* test (*df*)	—		1.470 (18)		0.306 (18)	1.409 (18)		1.409 (18)	
		*P* value	—		.16		.76	.18		.18	
	**VS^f^**										
		µ	1.417		1.407		1.387	1.395		1.372	
		σ	0.002		0.003		0.003	0.003		0.003	
		*t* test (*df*)	—		9.729 (18)		24.385 (18)	19.278 (18)		34.668 (18)	
		*P* value	—		<.001		<.001	<.001		<.001	
	***d***_**hellinger**_										
		µ	0.339		0.363		0.380	0.381		0.360	
		σ	0.009		0.008		0.018	0.011		0.018	
		*t* test (*df*)	—		6.589 (18)		6.375 (18)	9.481 (18)		3.217 (18)	
		*P* value	—		<.001		<.001	<.001		.005	
	**DD-plot ^g^ *R* ^2^**										
		µ	0.971		0.980		0.955	0.956		0.983	
		σ	0.005		0.003		0.004	0.003		0.002	
		*t* test (*df*)	—		4.767 (18)		7.413 (18)	7.505 (18)		7.101 (18)	
		*P* value	—		<.001		<.001	<.001		<.001	
**Privacy**											
	**MIA^h^**										
		µ	0		0^i^		0^i^	0^i^		0^i^	
		σ	0		0		0	0		0	
		*t* test (*df*)	—		—		—	—		—	
		*P* value	—		—		—	—		—	
	**AIA^j^**										
		µ	0.031		0.031		0.028	0.031		0.027	
		σ	0.015		0.013		0.009	0.016		0.018	
		*t* test (*df*)			0.063 (18)		0.422 (18)	0.024 (18)		0.559 (18)	
		*P* value			.95		.68	.98		.58	
	**Linkability**										
		µ	0.002		0.004		0.001	0.002^k^		0.003	
		σ	0.004		0.005		0.003	0.004		0.006	
		*t* test (*df*)	—		0.949 (18)		0.600 (18)	—		0.397 (18)	
		*P* value	—		.35		.55	—		.70	
	**Singling out**										
		µ	0.156		0.149		0.132	0.134		0.138	
		σ	0.040		0.031		0.040	0.031		0.044	
		*t* test (*df*)	—		0.425 (18)		1.287 (18)	1.344 (18)		0.922 (18)	
		*P* value	—		.68		.21	.20		.37	

^a^*t* tests were performed between the baseline and each federated experiment. Significance level is .05 for all statistical tests.

^b^CS: cosine similarity.

^c^Not applicable.

^d^DLA: data labeling analysis.

^e^AUC: area under the curve.

^f^VS: Vendi Score.

^g^DD-plot: depth versus depth plot.

^h^MIA: membership inference attack.

^i^*t* tests were not performed for these due to the standard deviation being zero.

^j^AIA: attribute inference attack.

^k^*t* tests were not performed for these due to the results being the same as the ones found in the baseline metrics.

In this scenario, the intervariable correlation metric, *ϕ*_k_, was the measure that most varied among the fidelity metrics, while the DLA metrics showed a similar tendency to the one found within the CTGAN results. The DD-plot *R*^2^ value, the VS, and the Hellinger distance, although statistically significant, did not show any relevant tendency linked to the number of nodes.

Regarding privacy measures, no membership inference risk was found among the generated SD in scenario B, while calculated linkability risks were minimal. Privacy risks did not significantly vary linked to the number of nodes.

In the IB scenario, the results were found to be similar to the ones found in scenario B, most of the fidelity metrics showing statistically significant differences between experiments with different numbers of nodes (*P*<.001), and no significance in privacy-related metrics (Table 6). In this scenario, the DLA metrics varied slightly more, even if no clear tendency is observable. In addition, the intervariable correlations seemed to be better maintained within this experiment, even if no tendency was found either.

**Table 6:** Fidelity and privacy metric results of FedTabDiff for the IB scenario^a^.

Metric				Baseline		3N	5N		7N		10N
**Fidelity**											
	**CS^b^*ϕ*_k_**										
		µ	0.866		0.632		0.838	0.857		0.531	
		σ	0.010		0.014		0.008	0.007		0.088	
		*t* test (*df*)	—^c^		41.539 (18)		6.657 (18)	2.318 (18)		11.294 (18)	
		*P* value	—		<.001		<.001	.03		<.001	
	**DLA^d^ AUC^e^**										
		µ	0.992		0.994		0.986	0.977		0.997	
		σ	0.001		0.001		0.001	0.001		0.002	
		*t* test (*df*)	—		6.063 (18)		10.205 (18)	35.140 (18)		15.540 (18)	
		*P* value	—		<.001		<.001	<.001		<.001	
	**DLA *F*_1_-score**										
		µ	0.997		0.999		0.995	0.992		0.999	
		σ	0.003		0.001		0.002	0.003		0.001	
		*t* test (*df*)	—		2.612 (18)		1.787 (18)	3.765 (18)		2.612 (18)	
		*P* value	—		.02		.09	.001		.02	
	**DLA recall**										
		µ	0.999		0.999		0.996	0.995		1^f^	
		σ	0.002		0.001		0.004	0.004		0	
		*t* test (*df*)	—		0.833 (18)		1.686 (18)	2.241 (18)		—	
		*P* value	—		.42		.11	.04		—	
	**VS^g^**										
		µ	1.417		1.422		1.408	1.404		1.394	
		σ	0.002		0.002		0.002	0.002		0.002	
		*t* test (*df*)	—		4.744 (18)		9.942 (18)	12.669 (18)		22.485 (18)	
		*P* value	—		<.001		<.001	<.001		<.001	
	***d***_**hellinger**_										
		µ	0.339		0.374		0.319	0.370		0.361	
		σ	0.009		0.029		0.003	0.003		0.013	
		*t* test (*df*)	—		3.525 (18)		6.849 (18)	10.258 (18)		4.280 (18)	
		*P* value	—		.002		<.001	<.001		<.001	
	**DD-plot** ^h^ ***R*** ^**2**^										
		µ	0.971		0.973		0.936	0.988		0.931	
		σ	0.005		0.005		0.007	0.001		0.005	
		*t* test (*df*)	—		0.739 (18)		11.702 (18)	9.648 (18)		17.166 (18)	
		*P* value	—		.47		<.001	<.001		<.001	
**Privacy**											
	**MIA^i^**										
		µ	0		0^f^		0^f^	0^f^		0^f^	
		σ	0		0		0	0		0	
		*t* test (*df*)	—		—		—	—		—	
		*P* value	—		—		—	—		—	
	**AIA^j^**										
		µ	0.031		0.021		0.019	0.020		0.024	
		σ	0.015		0.008		0.013	0.006		0.012	
		*t* test (*df*)	—		1.668 (18)		1.750 (18)	1.605 (18)		1.140 (18)	
		*P* value	—		.11		.10	.13		.27	
	**Linkability**										
		µ	0.002		0.004		0.001	0		0^f^	
		σ	0.004		0.010		0.003	0		0	
		*t* test (*df*)			0.638 (18)		0.600 (18)	0.939 (18)		—	
		*P* value			.53		.56	.36		—	
	**Singling out**										
		µ	0.156		0.156		0.133	0.134		0.142	
		σ	0.040		0.038		0.056	0.041		0.033	
		*t* test (*df*)	—		0.032 (18)		0.988 (18)	1.122 (18)		0.830 (18)	
		*P* value	—		.97		.34	.28		.42	

^a^*t* tests were performed between the baseline and each federated experiment. Significance level is .05 for all instances.

^b^CS: cosine similarity.

^c^Not applicable.

^d^DLA: data labeling analysis.

^e^AUC: area under the curve.

^f^*t* tests were not performed for these due to the standard deviation being zero.

^g^VS: Vendi Score.

^h^DD-plot: depth versus depth plot.

^i^MIA: membership inference attack.

^j^AIA: attribute inference attack.

Regarding the privacy metrics, no statistical significance was detected among the results. No membership inference risk was found in any of the experiments, while the linkability risk was minimal, as it was in scenario B.

The IB_non-iid_ scenario followed the same pattern as those of the B and IB scenarios, with the difference of some singling out risk differences were statistically significant in this case. No membership inference risk was found in this scenario, and the linkability risks were also found to be minimal (Table 7).

**Table 7:** Fidelity and privacy metric results of FedTabDiff for the IBnon-iid scenario^a^.

Metric				Baseline		3N	5N		7N		10N
**Fidelity**											
	**CS^b^*ϕ*_k_**										
		µ	0.866		0.613		0.529	0.570		0.545	
		σ	0.010		0.009		0.084	0.084		0.026	
		*t* test (*df*)	—^c^		56.766 (18)		12.014 (18)	10.579 (18)		34.233 (18)	
		*P* value	—		<.001		<.001	<.001		<.001	
	**DLA^d^ AUC^e^**										
		µ	0.992		0.995		0.998	0.997		0.998	
		σ	0.001		0.001		0.001	0.001		0.001	
		*t* test (*df*)	—		8.232 (18)		16.336 (18)	15.255 (18)		19.792 (18)	
		*P* value	—		<.001		<.001	<.001		<.001	
	**DLA *F*_1_-score**										
		µ	0.997		0.999		1^f^	0.999		1^f^	
		σ	0.003		0.001		0	0.001		0	
		*t* test (*df*)	—		2.612 (18)		—	2.612 (18)		—	
		*P* value	—		.02		—	.02		—	
	**DLA recall**										
		µ	0.999		1^f^		1^f^	1^f^		1^f^	
		σ	0.002		0		0	0		0	
		*t* test (*df*)	—		—		—	—		—	
		*P* value	—		—		—	—		—	
	**VS^g^**										
		µ	1.417		1.426		1.432	1.422		1.398	
		σ	0.002		0.002		0.001	0.001		0.002	
		*t* test (*df*)	—		9.581 (18)		18.524 (18)	5.557 (18)		19.534 (18)	
		*P* value	—		<.001		<.001	<.001		<.001	
	***d*_hellinger_**										
		µ	0.339		0.363		0.385	0.384		0.381	
		σ	0.009		0.006		0.018	0.014		0.003	
		*t* test (*df*)	—		6.171 (18)		7.107 (18)	8.471 (18)		15.022 (18)	
		*P* value	—		<.001		<.001	<.001		<.001	
	**DD-plot ^h^ *R* ^2^**										
		µ	0.971		0.947		0.578	0.931		0.913	
		σ	0.005		0.005		0.041	0.008		0.002	
		*t* test (*df*)	—		9.549 (18)		28.846 (18)	12.677 (18)		32.593 (18)	
		*P* value	—		<.001		<.001	<.001		<.001	
**Privacy**											
	**MIA^i^**										
		µ	0		0^f^		0^f^	0^f^		0^f^	
		σ	0		0		0	0		0	
		*t* test (*df*)	—		—		—	—		—	
		*P* value	—		—		—	—		—	
	**AIA^j^**										
		µ	0.031		0.028		0.020	0.027		0.038	
		σ	0.015		0.010		0.008	0.009		0.016	
		*t* test (*df*)	—		0.443 (18)		1.988 (18)	0.721 (18)		0.953 (18)	
		*P* value	—		.66		.06	.48		.35	
	**Linkability**										
		µ	0.002		0^f^		0.002^k^	0.001		0.005	
		σ	0.004		0		0.004	0.003		0.009	
		*t* test (*df*)	—		—		—	0.532 (18)		0.898 (18)	
		*P* value	—		—		—	.60		.38	
	**Singling out**										
		µ	0.156		0.099		0.101	0.087		0.091	
		σ	0.040		0.034		0.037	0.030		0.026	
		*t* test (*df*)	—		3.332 (18)		3.099 (18)	4.229 (18)		4.194 (18)	
		*P* value	—		.003		.006	<.001		<.001	

^a^*t* tests were performed between the baseline and each federated experiment. Significance level is .05 for all statistical tests.

^b^CS: cosine similarity.

^c^Not applicable.

^d^DLA: data labeling analysis.

^e^AUC: area under the curve.

^f^*t* tests were not performed for these due to the standard deviation being zero.

^g^VS: Vendi Score.

^h^DD-plot: depth versus depth plot.

^i^MIA: membership inference attack.

^j^AIA: attribute inference attack.

^k^*t* tests were not performed for these due to the results being the same as the ones found in the baseline metrics.

Fidelity metrics specifically showed a kind of worsening tendency in terms of the CS *ϕ*_k_ metric along with the increase in the number of nodes. In addition, the Hellinger distance seemed to increase accordingly. The DLA did not show any significant change, nor did the DD-plot *R*^2^.

**Figure 4:**
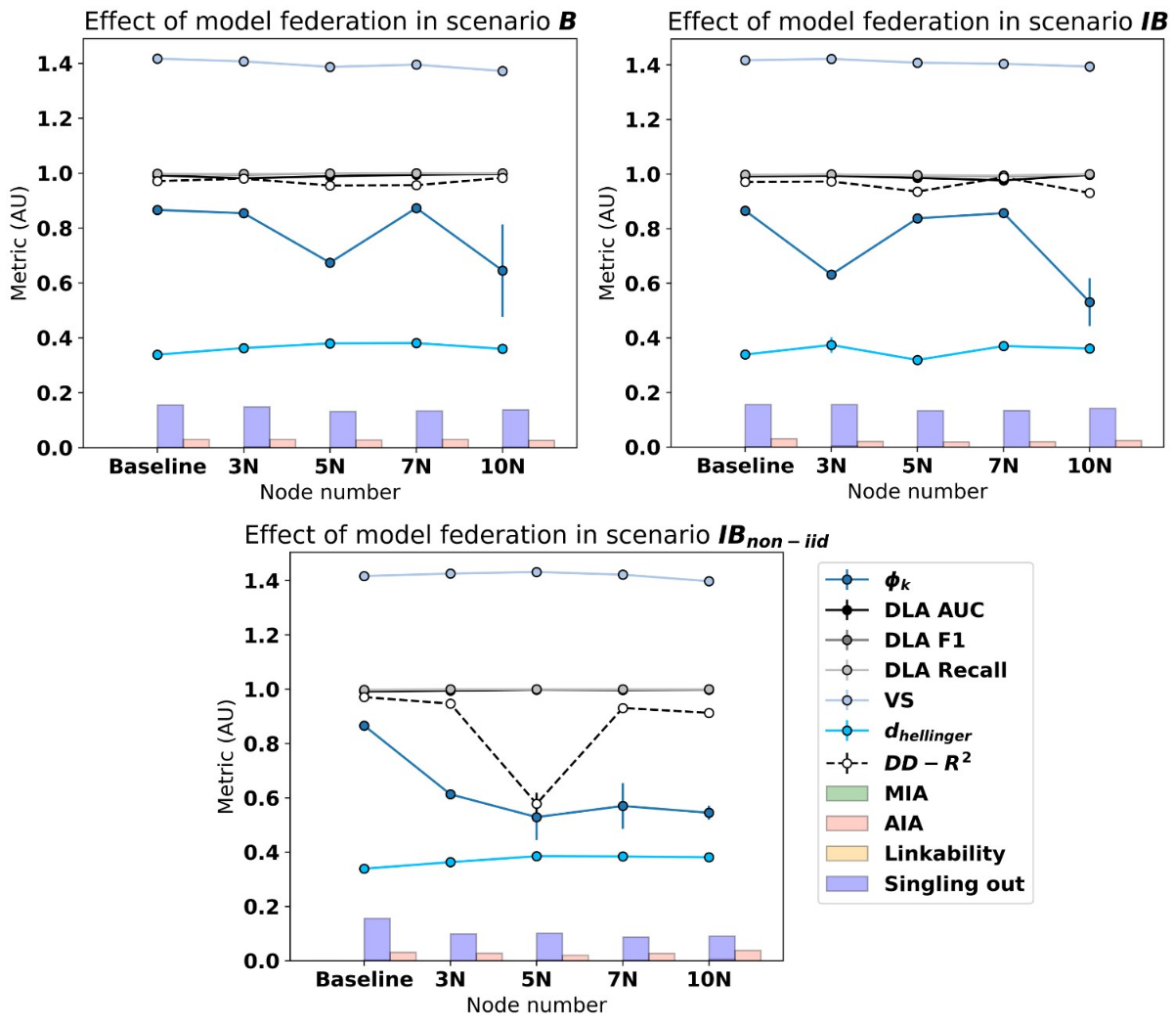
Graphical comparison of federated scenarios regarding fidelity and privacy metrics using the FedTabDiff model. AIA: attribute inference attack; AUC: area under the curve; DD: depth versus depth; DLA: data labeling analysis; MIA: membership inference attack; non-IID: nonindependent and identically distributed; VS: Vendi Score.

Now, gathering all the results in a single figure (Figure 4) to find all the information in a graphical manner, it can be observed that the singling out and attribute inference risks prevail over the membership and the linkability ones. Saying that, either a neutral tendency to maintain the privacy levels on the federated experiments, or an improvement of it (mostly in scenario IB_non-iid_), is observable with respect to the centralized baseline metrics.

As it was pointed out in the CTGAN results, the fidelity metric tendency can be found to kind of worsen once the FedTabDiff model is federated, but the effect seems to be mostly maintained across experimentation with different numbers of nodes.

Now, to finish with the result explanation and considering federated experiment pairs as previously (ie, comparing 3N experiments with 5N, 5N with 7N, and 7N with 10N), no statistically significant differences were observed in terms of the privacy metrics in any of the scenarios. Statistical significance was found in some of the comparisons across fidelity metrics and different scenarios (Table 8).

**Table 8:** *t* test results for FedTabDiff experiment pairs^a^.

Metric				3N-5N				5N-7N				7N-10N		
				*t* test (*df*)		*P* value		*t* test (*df*)		*P* value		*t* test (*df*)		*P* value
**B scenario**														
	**Fidelity**													
		CS^b^ *ϕ*_k_	2.917 (18)		.009		3.230 (18)		.005		4.051 (18)		<.001	
		DLA^c^ AUC^d^	14.827 (18)		<.001		13.258 (18)		<.001		13.524 (18)		<.001	
		DLA *F*_1_-score^e^	5.701 (18)		<.001		—^f^		—		—		—	
		DLA recall^g^	1.849 (18)		.08		—		—		—		—	
		VS^h^	14.140 (18)		<.001		5.787 (18)		<.001		16.078 (18)		<.001	
		*d*_hellinger_	2.780 (18)		.01		0.202 (18)		.84		3.176 (18)		.005	
		DD-plot^i^ *R*^2^	15.341 (18)		<.001		0.469 (18)		.64		21.397 (18)		<.001	
	**Privacy**													
		MIA^g,j^	—		—		—		—		—		—	
		AIA^k^	0.560 (18)		.58		0.356 (18)		.73		0.508 (18)		.62	
		Linkability	1.567 (18)		.13		0.600 (18)		.56		0.397 (18)		.70	
		Singling out	0.985 (18)		.34		0.094 (18)		.93		0.234 (18)		.82	
**IB scenario**														
	**Fidelity**													
		CS *ϕ*_k_	39.051 (18)		<.001		5.080 (18)		<.001		11.001 (18)		<.001	
		DLA AUC	16.701 (18)		<.001		18.768 (18)		<.001		80.136 (18)		<.001	
		DLA *F*_1_-score	6.293 (18)		<.001		2.528 (18)		.02		7.400 (18)		<.001	
		DLA recall^g^	2.472 (18)		.02		0.617 (18)		.54		—		—	
		VS	14.454 (18)		<.001		3.620 (18)		.002		8.573 (18)		<.001	
		*d*_hellinger_	5.667 (18)		<.001		32.574 (18)		<.001		2.082 (18)		.05	
		DD-plot *R*^2^	11.921 (18)		<.001		20.719 (18)		<.001		34.050 (18)		<.001	
	**Privacy**													
		MIA^a^	—		—		—		—		—		—	
		AIA	0.400 (18)		.69		0.072 (18)		.94		0.873 (18)		.39	
		Linkability^g^	0.946 (18)		.36		—		—		—		—	
		Singling out	1.016 (18)		.32		0.153 (18)		.88		0.686 (18)		.50	
**IB_non-iid_ scenario**														
	**Fidelity**													
		CS *ϕ*_k_	3.011 (18)		.007		1.036 (18)		.31		0.834 (18)		.42	
		DLA AUC	10.399 (18)		<.001		2.494 (18)		.02		7.239 (18)		<.001	
		DLA *F*_1_-score^g^	—		—		—		—		—		—	
		DLA recall^g^	—		—		—		—		—		—	
		VS	8.510 (18)		<.001		16.032 (18)		<.001		27.498 (18)		<.001	
		*d*_hellinger_	3.310 (18)		.004		0.221 (18)		.83		0.446 (18)		.66	
		DD-plot *R*^2g^	27.073 (18)		<.001		25.599 (18)		<.001		7.204 (18)		<.001	
	**Privacy**													
		MIA^g^	—		—		—		—		—		—	
		AIA	2.029 (18)		.06		1.702 (18)		.11		1.788 (18)		.09	
		Linkability^e,g^	—		—		0.532 (18)		.60		0.898 (18)		.38	
		Singling out	0.082 (18)		.94		1.224 (18)		.24		0.736 (18)		.47	

^a^Significance level is .05 for all statistical tests.

^b^CS: cosine similarity.

^c^DLA: data labeling analysis.

^d^AUC: area under the curve.

^e^*t* tests were not performed for these due to the results being the same as the ones found in the baseline metrics.

^f^Not applicable.

^g^*t* tests were not performed for these due to the standard deviation being zero.

^h^VS: Vendi Score.

^i^DD-plot: depth versus depth plot.

^j^MIA: membership inference attack.

^k^AIA: attribute inference attack.

## Discussion

### Principal Results

In these experiments, a comparison between centralized SDG models and federated implementations of them was performed using AML data with the aim of evaluating the SD fidelity and privacy in each of the scenarios, assessing the SD generation techniques over an FL approach to address the data scattering issue while addressing data scarcity. Three different scenarios were considered for the federated models: the one in which the number of samples in each node was evenly distributed (B), the one where the node-wise data quantity was randomly and unevenly distributed (IB), and the one where non-IID data distributions were created (IB_non-iid_).

In the case of the CTGAN model, in the B scenario, the *ϕ*_k_ metric deteriorated to a maximum of 9% with respect to the baseline, while the DLA showed an average difference of 17% in the AUC, a difference of 9% for the *F*_1_-score, and a difference of 0.21% for the recall metric. The VS showed a difference between 2% and 12% showing that the diversity of the generated samples varied among experiments. For this scenario, the Hellinger distance varied to a maximum of 3%, and the DD-plot *R*^2^ to a maximum of 35%. The most privacy-preserving experiments in terms of the AIA were 3N and 7N, with a risk reduction of 18% with respect to the baseline, while the worst one (5N) performed 18% below, although not statistically significant. Privacy against singling out attacks improved between 46% and 61%, while no difference was found for MIAs and linkability attacks.

In the IB scenario, the maximum deterioration of the *ϕ*_k_ metric was 11%, and the DLA showed average values of 17% in the case of the AUC, 10% for the *F*_1_-score, and 1% for the recall metric. The VS showed that numerical variables are diversely generated, achieving differences between 1% and 3%, while the Hellinger distances varied to a maximum of 3%. DD-plot *R*^2^ values varied by about 22%. AIA analyses showed a maximum improvement of 55% on data privacy, while the maximum deterioration was 26%, although not statistically significant. Singling out attacks showed differences between 51% and 80%, while the linkability ones varied minimally.

Finally, the IB_non-iid_ scenario showed a maximum deterioration of 11% in the *ϕ*_k_ metric, while DLA AUC scores showed average differences of 21%, the *F*_1_-score varied 10%, and the recall varied minimally. The VS showed maximum variations of 17%, and the Hellinger distance varied to 5%. Regarding privacy metrics, the AIA showed a maximum improvement of 2% and a maximum deterioration of 21%, although neither was statistically significant in this case either. In this case, singling out attack risk differences varied between 73% and 83% with respect to the baseline, and the linkability measure differences were again minimal.

Now talking about the principal results of the FedTabDiff model in scenario B, the metric varied between 26% and 81%, the VS between 1% and 3%, and the Hellinger distance between 6% and 12%. In the DLA, the AUC value varied to a maximum of 1% while both the recall and the *F*_1_-score matched the baseline results. To finish with the fidelity metrics, the DD-plot *R*^2^ results varied between 1% and 2% with respect to the baseline. In terms of privacy, no membership inference risk was detected, while the linkability risk difference was minimal. The AIA results varied between 10% and 13%, while the singling out ones varied between 4% and 15%.

Talking about scenario IB, the *ϕ*_k_ metric varied between 1% and 27%, and the DLA-related AUC metric varied by 2% at most, matching the baseline results for both the recall and the *F*_1_-score, just as in the B scenario. The VS variation was neither high, reaching 2% differences at most. While the Hellinger distance oscillated between 6% and 10%, the DD-plot *R*^2^ varied to a maximum of 4%. Regarding the privacy metrics, no membership inference risk was found, and the linkability varied minimally with respect to the baseline results. While the attribute inference risk difference oscillated between 23% and 39%, the singling out risk difference varied to a maximum of 15%.

To finish, the IB_non-iid_ scenario showed an oscillation between 29% and 39% for the *ϕ*_k_ metric, while all the DLA AUC changes were minimal and the recall- and *F*_1_-score-related results matched those of the baseline. For the VS, the variation reached 22% at maximum, and the Hellinger distance varied between 7% and 62%. The DD-plot *R*^2^ varied between 2% and 40%. To finish, the privacy metrics in this scenario followed the previous tendencies by showing no membership inference risk and minimal linkability risks. Attribute inference risk differences oscillated between 10% and 35%, while the singling out ones varied between 35% and 44%.

### Limitations

As it was pointed out in the beginning, this work aims to provide insight into a specific use case of federated SDG for an AML dataset, both with a GAN-type model and a diffusion-type model. However, extending the analysis by using upcoming SDG models and more extensive aggregation functions for the FL framework may result in more generalizable conclusions, which will be prioritized in future work. Linked with that, models incorporating more novel tools like DP and the implementation of advanced FL security frameworks should be covered in future extensions of this research. Parameter tuning for model optimization remains a considerable path for analyzing the combination of both SDG and FL.

Furthermore, the three scenarios resulted in similar overall tendencies for all the calculated metrics and both models, suggesting the scenario proposals in this work may not have that much of an impact on the results. Further research may uncover differences for various data dispersion schemas and non-IID distribution types, which may have a much greater impact on the calculated metrics and the methodology to be followed. In addition, extremely imbalanced approaches may show different tendencies in the analyses.

Finally, the generative aspect of this research should be taken into account for future research, as expanding the calculations to a higher number of datasets may result in more robust and scalable optimizations on federated SDG. Related to the issue, a more extensive set of metrics could also be considered in the future, as literature is rapidly evolving and novel metrics could be introduced in upcoming publications. Our metric selection, however, is intended to be comparable to other state-of-the-art works.

### Comparison With Prior Work

While there are a few studies that analyze the combination of both FL and SDG, this is, to the best of the authors’ knowledge, the first research work that tries to quantify the impact of generative model federation over fidelity and privacy metrics, using different numbers of nodes, and considering different real-world data distribution scenarios in AML.

Expanding the literature search, incorporating DP to federated SDG models has been widely investigated, as well as successfully integrated into several use cases, improving privacy metrics under those conditions [46-48]. Our research, however, has shown reasonable privacy guarantees both for centralized and federated scopes without the need for using DP, suggesting the incorporation of it may depend on the final use case, as it deteriorates SD fidelity, even though more extensive evaluations should be performed. In addition, the latest research shows DP may not have statistically significant differences in terms of privacy, suggesting that applications with no need to implement it exist [63].

Furthermore, in the scope of FL, previous works on classification and regression models have hardly shown any deterioration due to the federation process with regard to a centralized model [67]. Instead, when SDG models were compared to centralized results in this AML use case, statistically significant differences appeared, suggesting that SDG models may be more sensible to the federation step than usual ML cases, such as classification or regression.

To finish, the data distribution scenarios considered for this research demonstrated robustness against non-IID distributions, which is in line with other experiments performed in the literature [68].

### Conclusions

The results for both models and the three scenarios showed considerable data fidelity losses after the model federation process, while no significant deterioration or improvement tendency was found in the respective privacy metrics. The number of federated nodes did not show any significant trend, even though specific comparisons resulted in statistically relevant differences in some cases.

## Appendix

Multimedia Appendix 1 Description of the variables that were included in this study.

Multimedia Appendix 2 Categorical variable distributions and number of samples per node and scenario.

Multimedia Appendix 3 Aggregation algorithm comparison.

Multimedia Appendix 4 Variable-wise metric results using the CTGAN model.

Multimedia Appendix 5 Variable-wise metric results using the FedTabDiff model.

## Abbreviations

AI: artificial intelligence
AIA: attribute inference attack
AML: acute myeloid leukemia
AUC: area under the curve
CS: cosine similarity
CTGAN: conditional tabular generative adversarial network
DD-plot: depth versus depth plot
DLA: data labeling analysis
DP: differential privacy
EHR: electronic health record
FL: federated learning
GAN: generative adversarial network
HFL: horizontal federated learning
MIA: membership inference attack
ML: machine learning
Non-IID: nonindependent and identically distributed
SD: synthetic data
SDG: synthetic data generation
VAE: variational autoencoder
VS: Vendi Score
WHO: World Health Organization

## Data Availability

The original datasets analyzed during this study are available in the Tazi_NatureC_AML repository [49,50].
