# Supplementary material for: Synthetic Tabular Data Generation Under Horizontal Federated Learning Environments in Acute Myeloid Leukemia: Case-Based Simulation Study
Source: JMIR Med Inform. 2025 Sep 29;13:e74116. doi: 10.2196/74116 (PMC12519032; doi:10.2196/74116)
Supplement: Multimedia Appendix 1 [file medinform_v13i1e74116_app1.pdf]

| Variable | Type | Description |
|---|---|---|
| **Clinical** | | |
| Age (years) | Continuous | Age of the patient |
| BM_blasts (%) | Continuous | Number of bone marrow blasts |
| HB (g/dl) | Continuous | Hemoglobin |
| PLT ($10^{-9}$/L) | Continuous | Platelet count |
| WBC ($10^{-9}$/L) | Continuous | Number of white blood cells |
| OS | Continuous | Overall survival |
| Perf_status (ECOG[a]) | Categorical | Performance status in the ECOG scale |
| AHD | Binary | Antecedent hematologic disease |
| OS_status | Binary | Overall survival status |
| **Demographic** | | |
| Gender | Binary | Gender of the patient |
| **Disease-related** | | |
| Secondary | Categorical | Secondary AML[b] |
| Eln_2017 | Categorical | European LeukemiaNet 2017 risk classification |

[a]ECOG: Eastern Cooperative Oncology Group.

[b]AML: acute myeloid leukemia.
