# Supplementary material for: Synthetic Tabular Data Generation Under Horizontal Federated Learning Environments in Acute Myeloid Leukemia: Case-Based Simulation Study
Source: JMIR Med Inform. 2025 Sep 29;13:e74116. doi: 10.2196/74116 (PMC12519032; doi:10.2196/74116)
Supplement: Multimedia Appendix 2 [file medinform_v13i1e74116_app2.pdf]

List of variables included:

Variable	Type	Description
**Clinical**		
Perf_status (ECOG)	Categorical	Performance status in Eastern Cooperative Oncology Group (ECOG) scale
AHD	Binary	Antecedent hematologic disease
OS_status	Binary	Overall survival status
**Demographic**		
Gender	Binary	Gender of the patient
**Disease-related**		
Secondary	Categorical	Secondary AML
Eln_2017	Categorical	European LeukemiaNet 2017 risk classification

Number of samples used for each experiment:

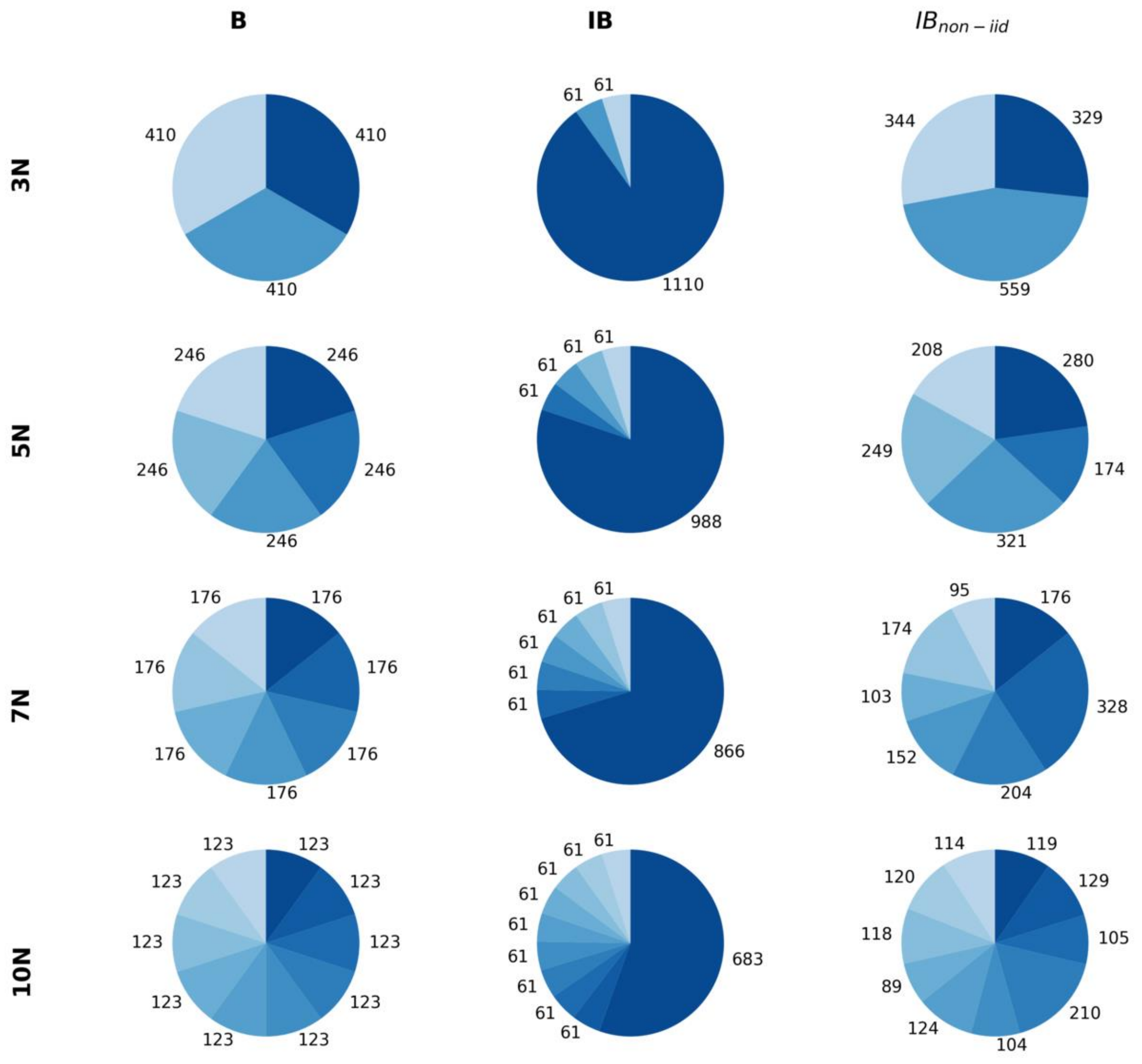

Distributions:

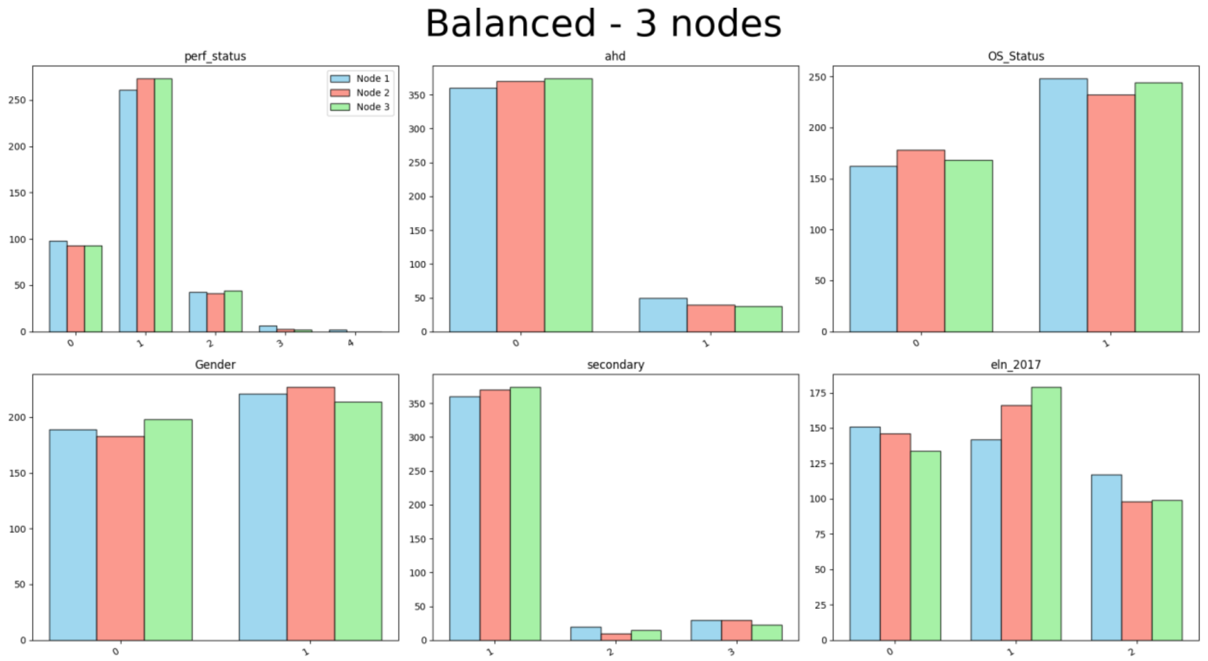

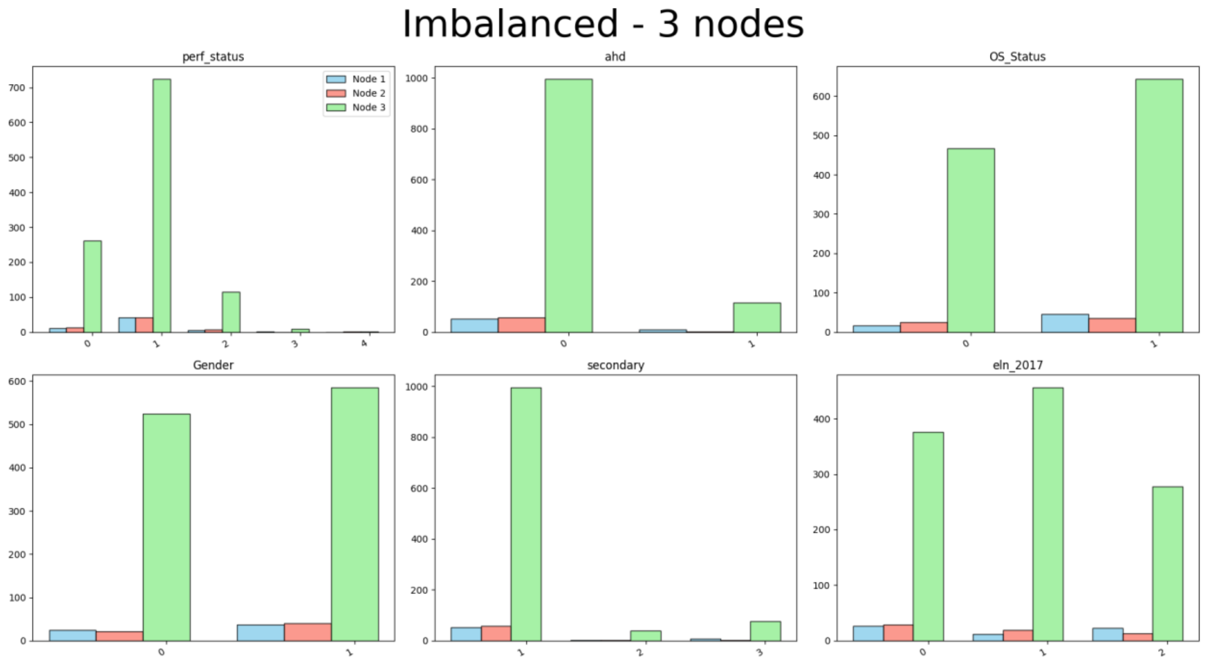

Imbalanced (non-IID) - 3 nodes

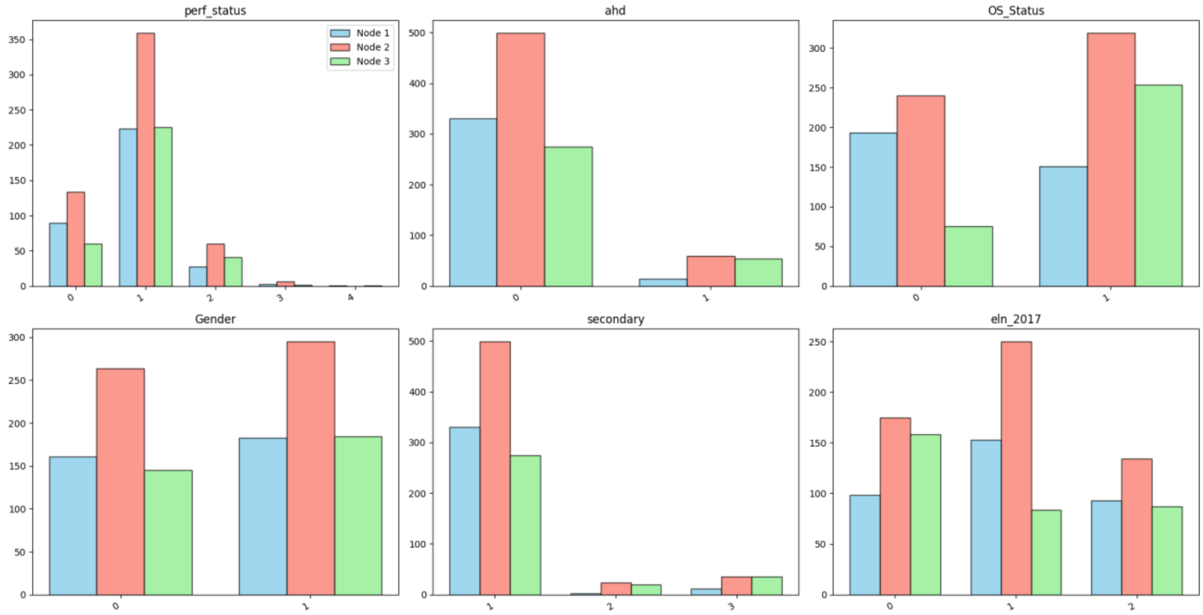

Balanced - 5 nodes

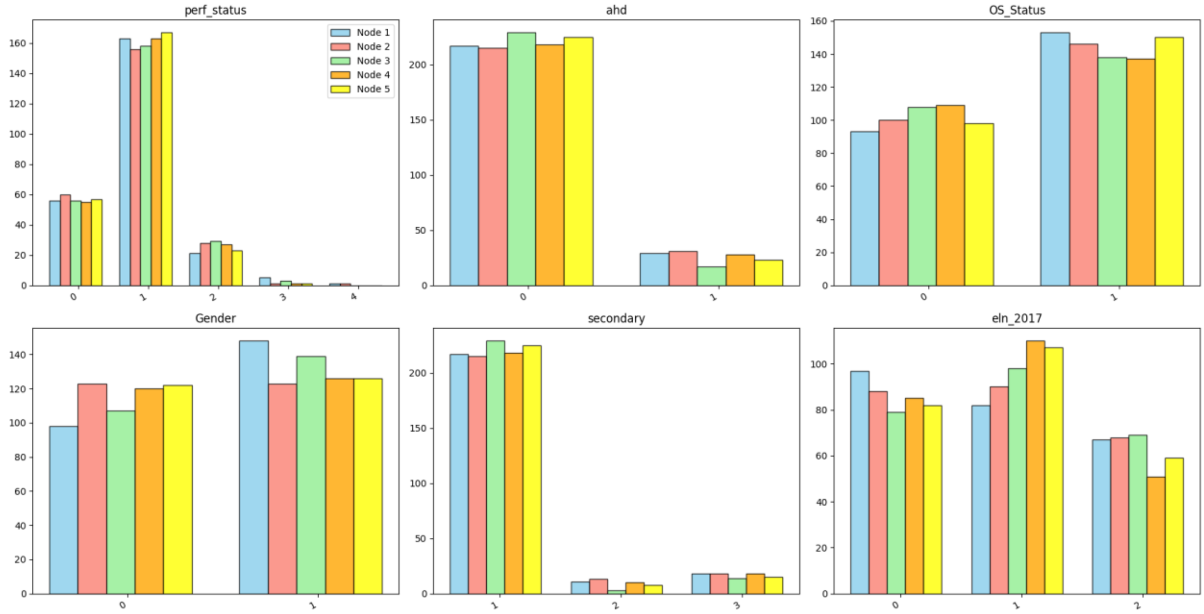

Imbalanced - 5 nodes

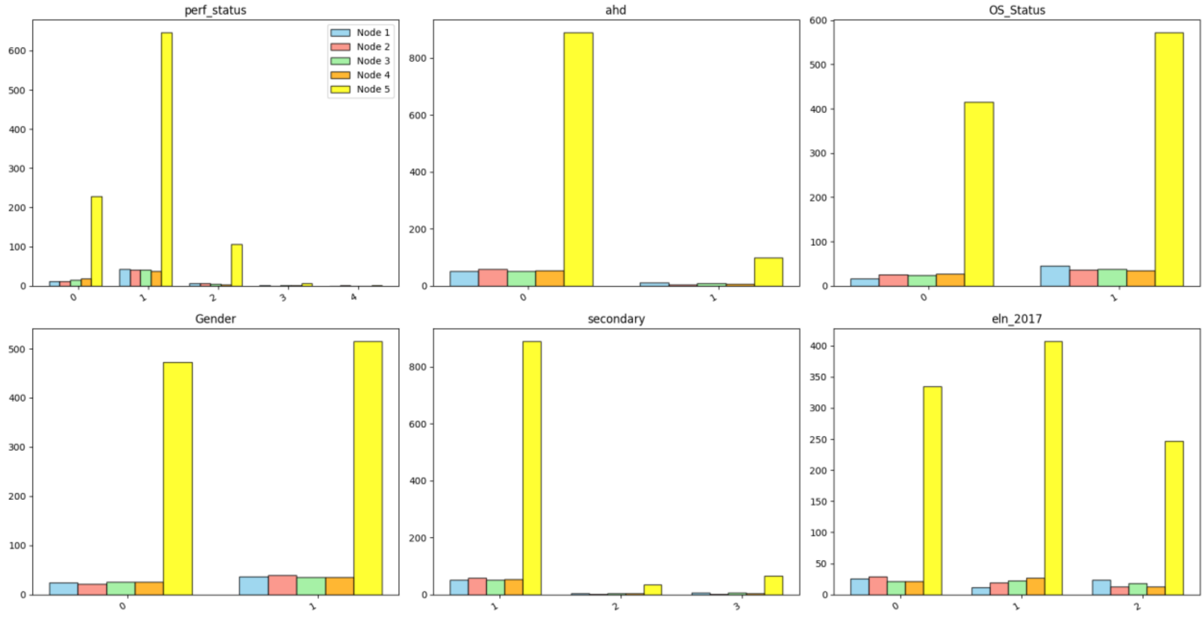

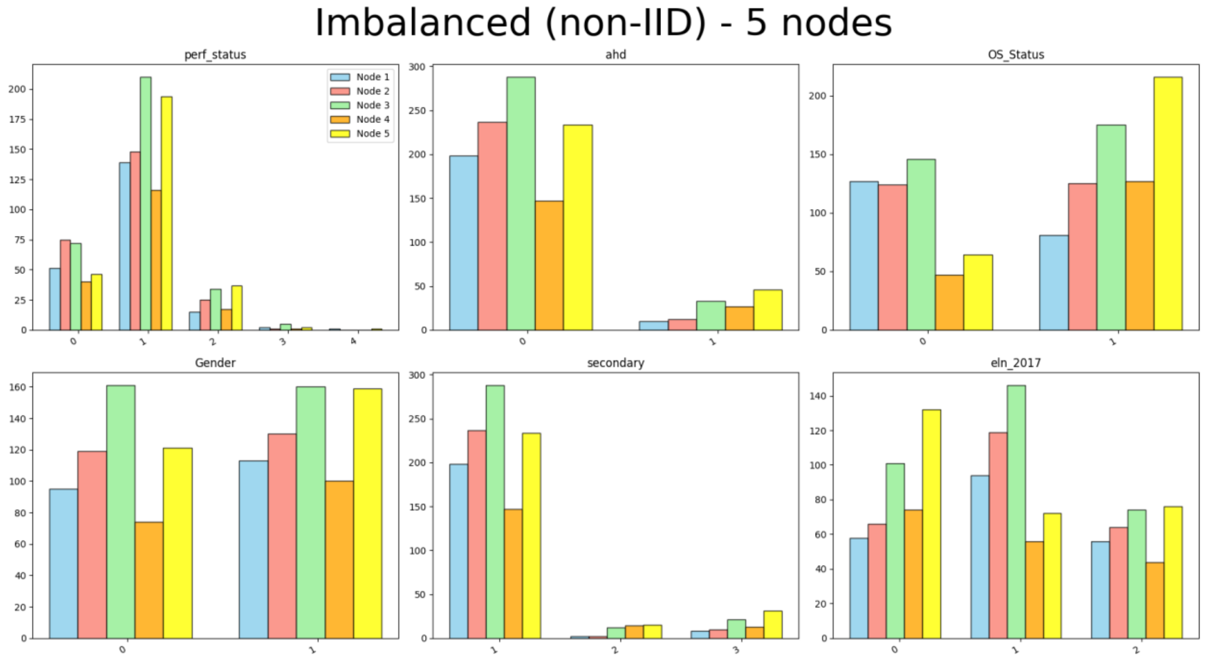
Imbalanced (non-IID) - 5 nodes
perf_status
ahd
OS_Status
Gender
secondary
eln_2017
Node 1
Node 2
Node 3
Node 4
Node 5

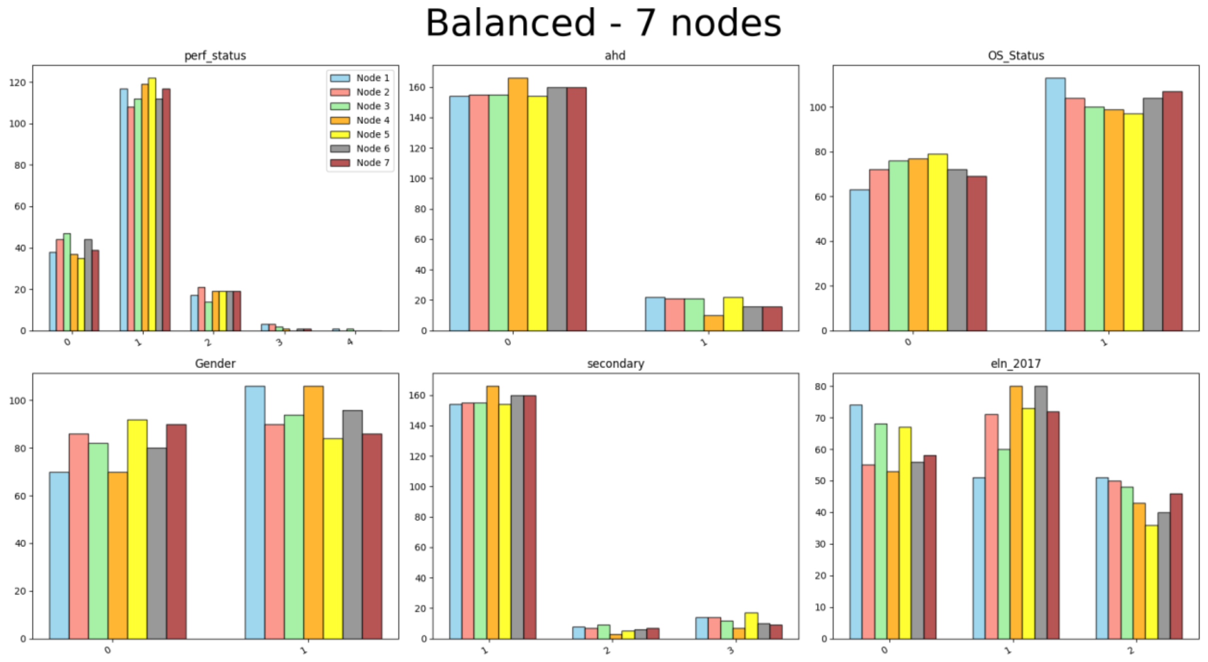
Balanced - 7 nodes
perf_status
ahd
OS_Status
Gender
secondary
eln_2017
Node 1
Node 2
Node 3
Node 4
Node 5
Node 6
Node 7

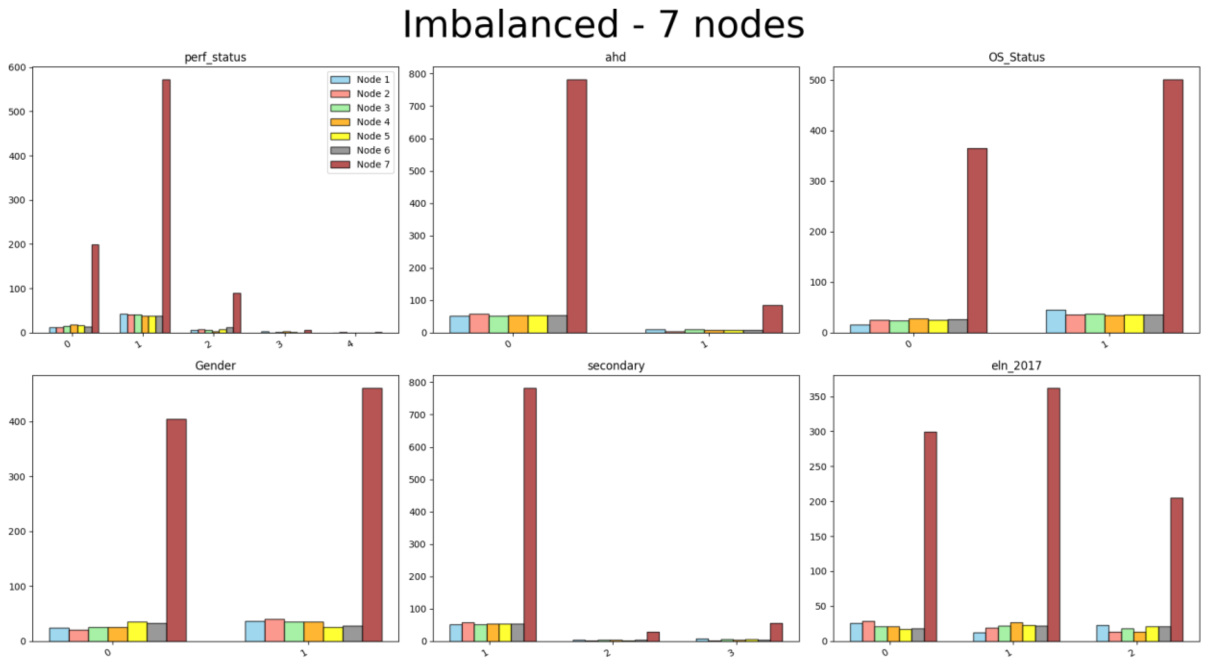
Imbalanced - 7 nodes
perf_status
ahd
OS_Status
Gender
secondary
eln_2017
Node 1
Node 2
Node 3
Node 4
Node 5
Node 6
Node 7

Imbalanced (non-IID) - 7 nodes

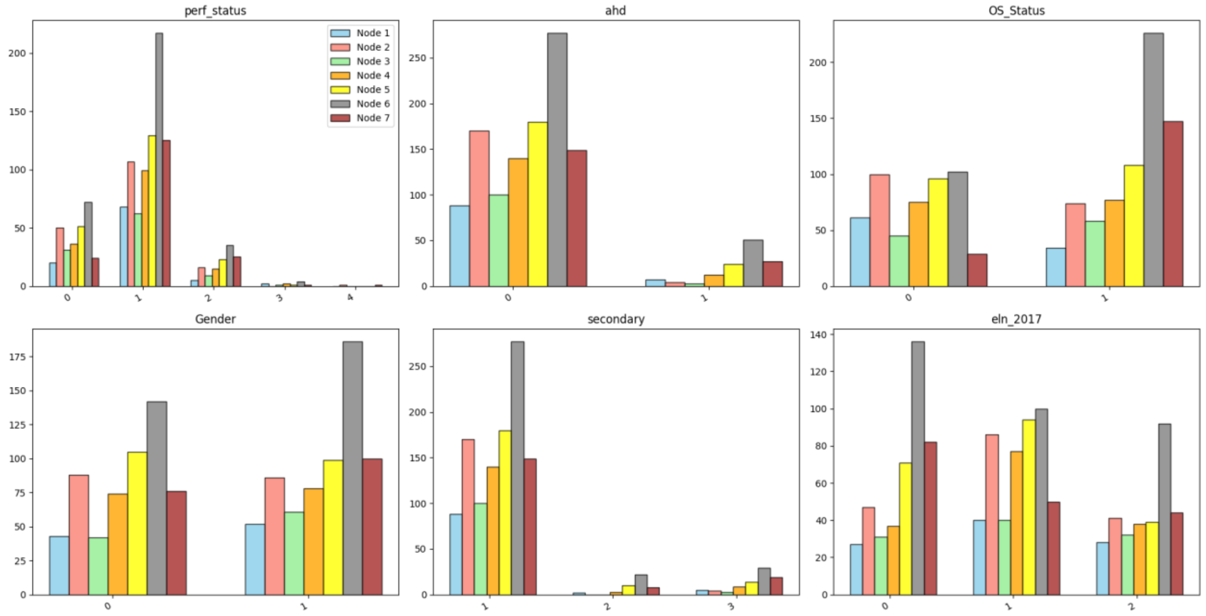

Balanced - 10 nodes

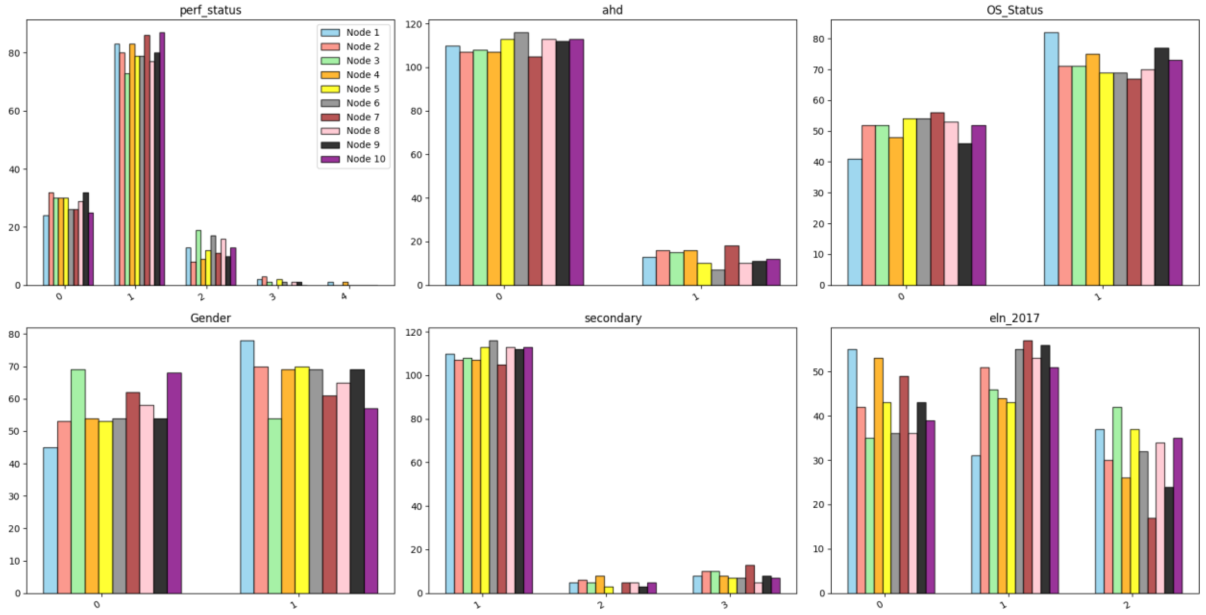

Imbalanced - 10 nodes

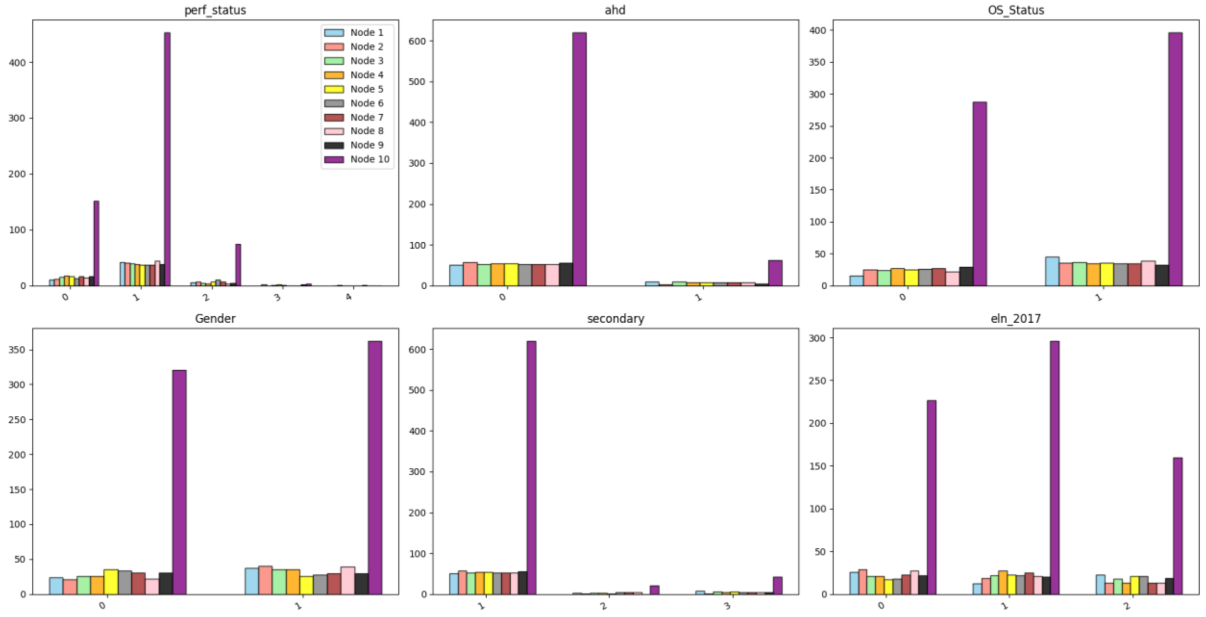

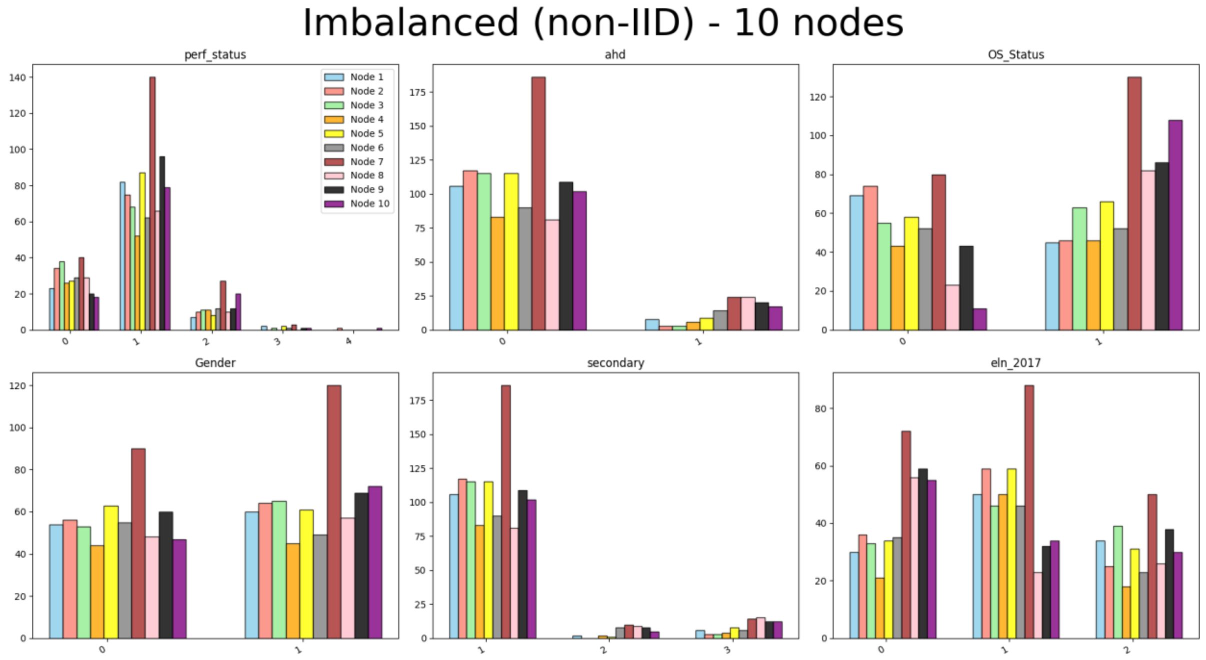
Imbalanced (non-IID) - 10 nodes
perf_status
ahd
OS_Status
Gender
secondary
eln_2017
Node 1
Node 2
Node 3
Node 4
Node 5
Node 6
Node 7
Node 8
Node 9
Node 10
