# Supplementary material for: Synthetic Tabular Data Generation Under Horizontal Federated Learning Environments in Acute Myeloid Leukemia: Case-Based Simulation Study
Source: JMIR Med Inform. 2025 Sep 29;13:e74116. doi: 10.2196/74116 (PMC12519032; doi:10.2196/74116)
Supplement: Multimedia Appendix 3 [file medinform_v13i1e74116_app3.pdf]

Metrics	CTGAN			FedTabDiff		
	FedAvg (baseline)	FedOpt	FedProx	FedAvg (baseline)	FedOpt	FedProx
**Fidelity**						
CS ϕ_k						
μ (σ)	0.842 (0.003)	0.825 (0.006)	0.821 (0.008)	0.545 (0.026)	0.620 (0.019)	0.696 (0.087)
$t_{0.05, 18}$		7.507	7.600		6.942	4.990
P		<.001	<.001		<.001	<.001
DLA AUC						
μ (σ)	0.950 (0.006)	0.915 (0.003)	0.895 (0.003)	0.998 (0.001)	0.998 (0.001)	0.998 (0.001)
$t_{0.05, 18}$		16.770	26.264		2.015	0.001
P		<.001	<.001		.059	.99
DLA F1						
μ (σ)	0.950 (0.006)	0.956 (0.008)	0.951 (0.010)	1 (0)	1 (0)	1 (0)
$t_{0.05, 18}$		1.927	0.487		-	-
P		.07	.63		-	-
DLA Recall						
μ (σ)	0.950 (0.004)	0.965 (0.016)	0.957 (0.013)	1 (0)	1 (0)	1 (0)
$t_{0.05, 18}$		1.871	0.874		-	-
P		.08	.40		-	-
VS						
μ (σ)	1.367 (0.004)	1.295 (0.005)	1.306 (0.007)	1.398 (0.002)	1.417 (0.001)	1.413 (0.002)
$t_{0.05, 18}$		34.494	23.326		21.846	14.323
P		<.001	<.001		<.001	<.001
$d_{hellinger}$						
μ (σ)	0.213 (0.004)	0.217 (0.002)	0.217 (0.002)	0.381 (0.003)	0.385 (0.010)	0.388 (0.006)
$t_{0.05, 18}$		4.329	4.107		1.100	0.014
P		<.001	<.001		.29	2.733
DD-plot R^2						
μ (σ)	0.847 (0.004)	0.842 (0.005)	0.832 (0.004)	0.913 (0.002)	0.838 (0.006)	0.680 (0.024)
$t_{0.05, 18}$		2.592	9.415		30.150	30.031
P		.02	<.001		<.001	<.001

Metrics	CTGAN			FedTabDiff		
	FedAvg (baseline)	FedOpt	FedProx	FedAvg (baseline)	FedOpt	FedProx
**Privacy**						
MIA						
μ (σ)	0 (0)	0 (0)	0 (0)	0 (0)	0(0)	0(0)
$t_{0.05, 18}$		-	-		-	-
P		-	-		-	-
AIA						
μ (σ)	0.057 (0.032)	0.028 (0.011)	0.032 (0.017)	0.038 (0.016)	0.026 (0.010)	0.025 (0.013)
$t_{0.05, 18}$		8.238	4.583		1.916	1.913
P		<.001	.001		.07	.07
Linkability						
μ (σ)	0 (0)	0.001 (0.003)	0 (0)	0.005 (0.009)	0 (0)	0.002 (0.006)
$t_{0.05, 18}$		-	-		-	0.806
P		-	-		-	.43
Singling out						
μ (σ)	0.021 (0.021)	0.031 (0.029)	0.023 (0.023)	0.091 (0.026)	0.077 (0.028)	0.089 (0.029)
$t_{0.05, 18}$		1.074	0.245		1.075	0.152
P		.31	.81		.30	.88
