# Supplementary material for: Synthetic Tabular Data Generation Under Horizontal Federated Learning Environments in Acute Myeloid Leukemia: Case-Based Simulation Study
Source: JMIR Med Inform. 2025 Sep 29;13:e74116. doi: 10.2196/74116 (PMC12519032; doi:10.2196/74116)
Supplement: Multimedia Appendix 4 [file medinform_v13i1e74116_app4.pdf]

1) AIA risk vs. Hellinger distance comparison

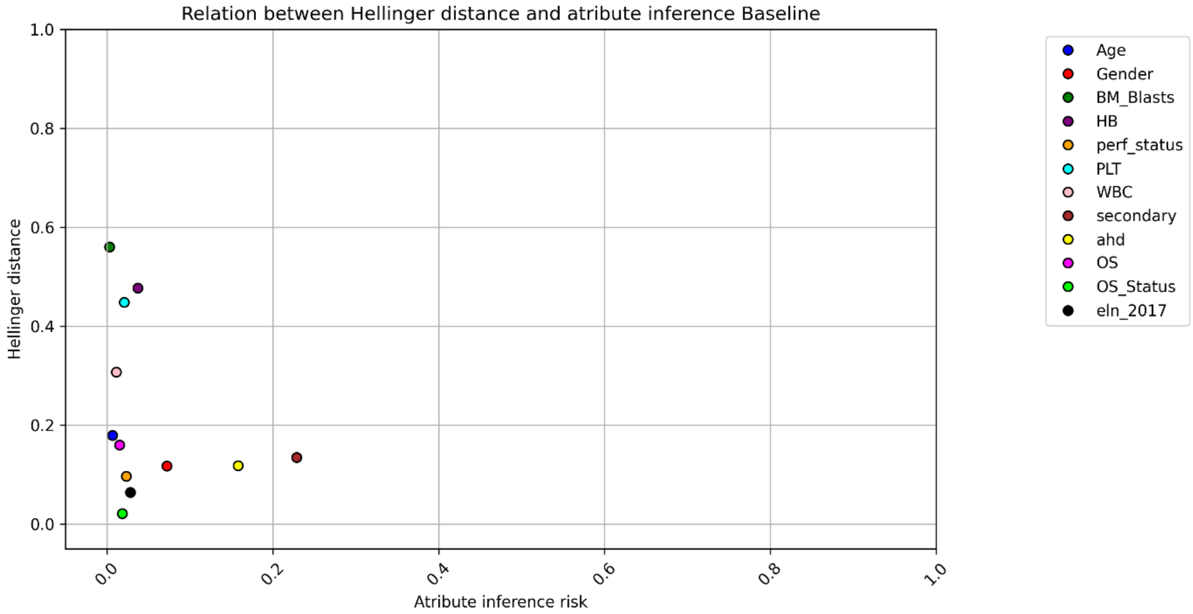

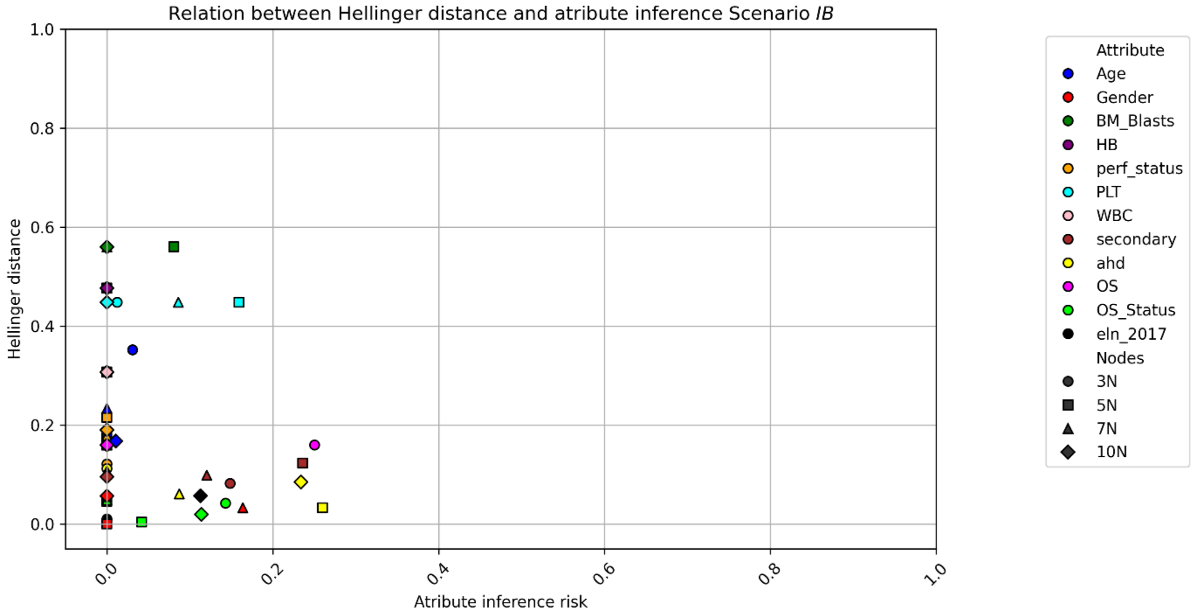

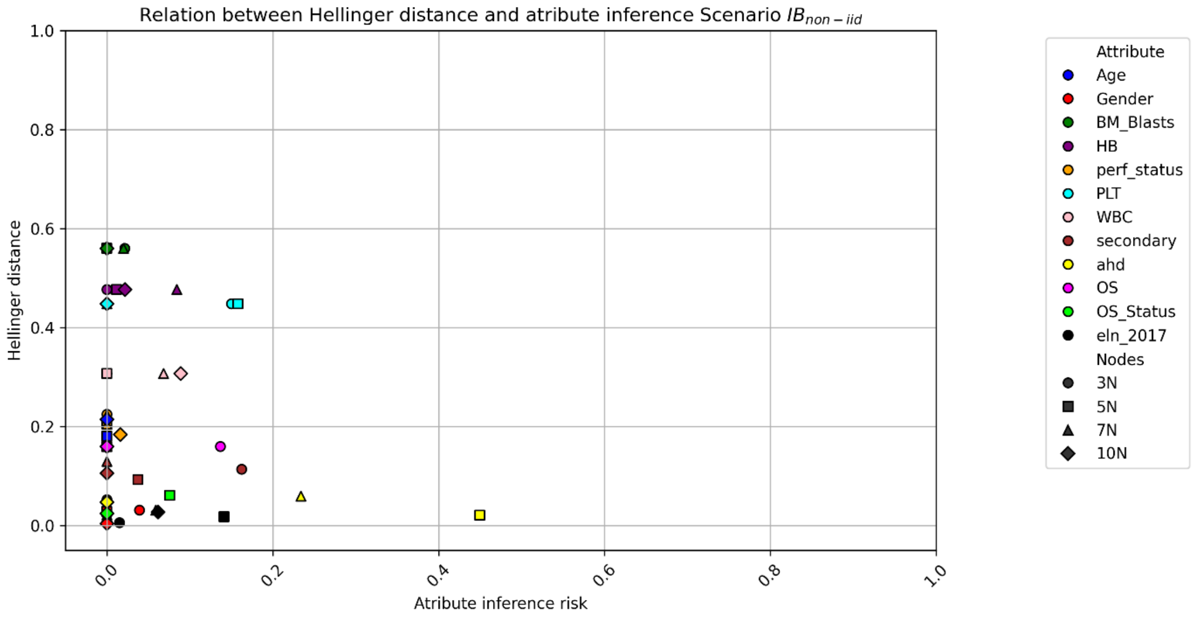

Relation between Hellinger distance and atribute inference Scenario $IB_{non-iid}$
Hellinger distance
Atribute inference risk
0.0
0.2
0.4
0.6
0.8
1.0
Attribute
Age
Gender
BM_Blasts
HB
perf_status
PLT
WBC
secondary
ahd
OS
OS_Status
eln_2017
Nodes
3N
5N
7N
10N

2) Hellinger distances variable-wise

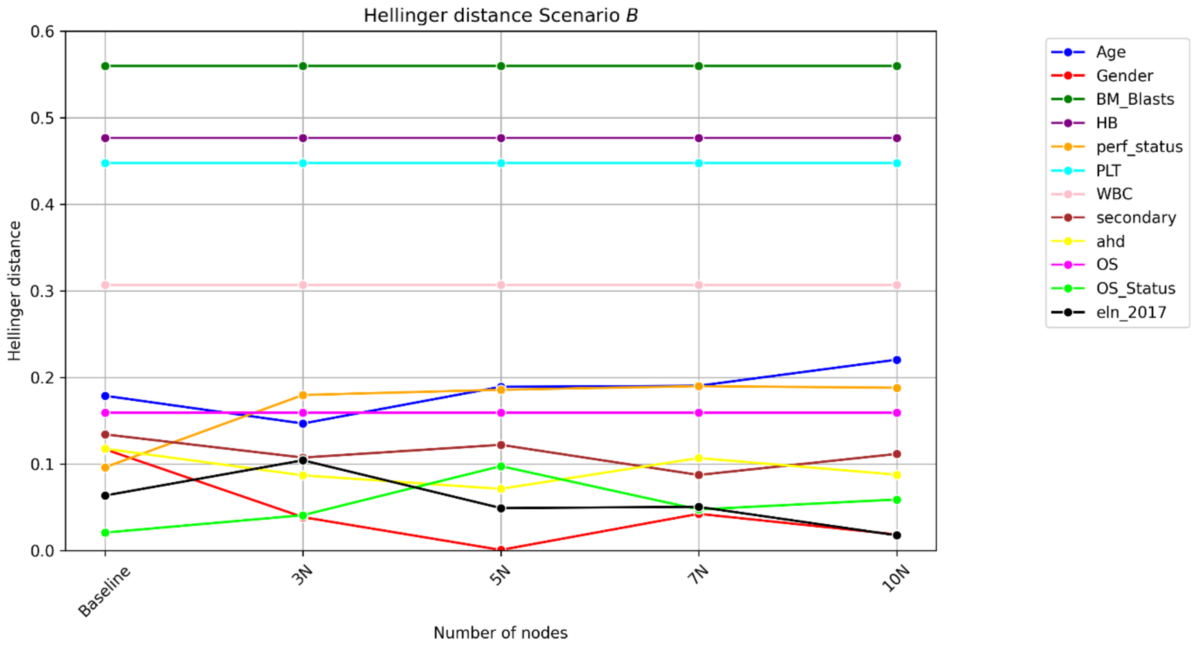

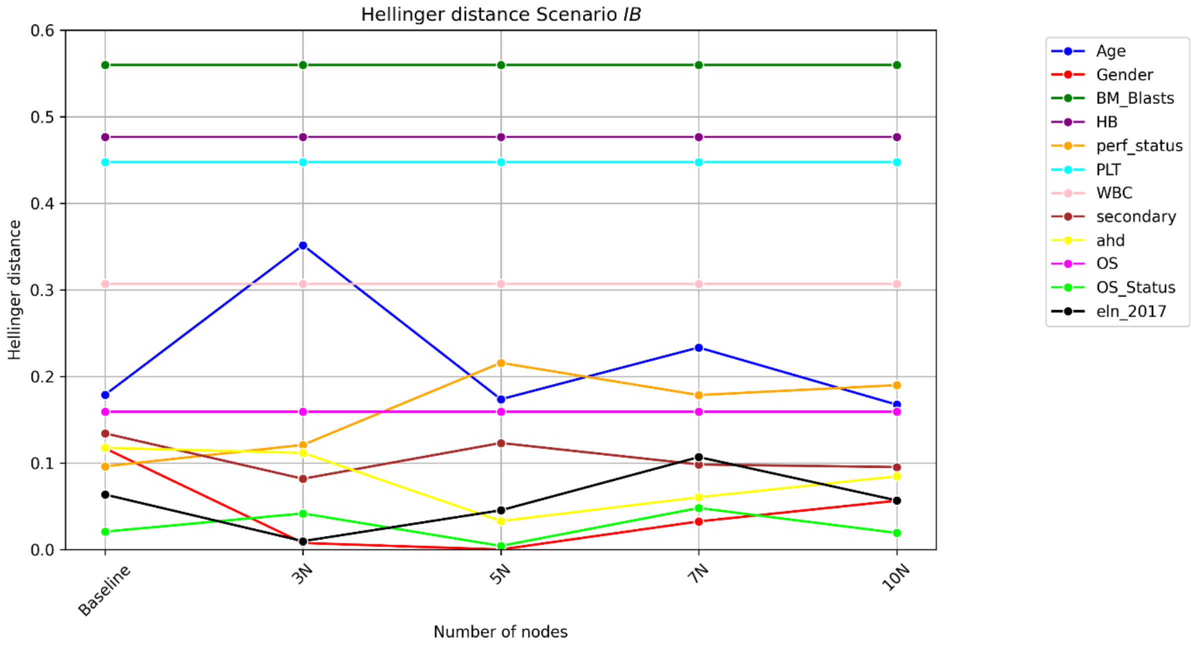

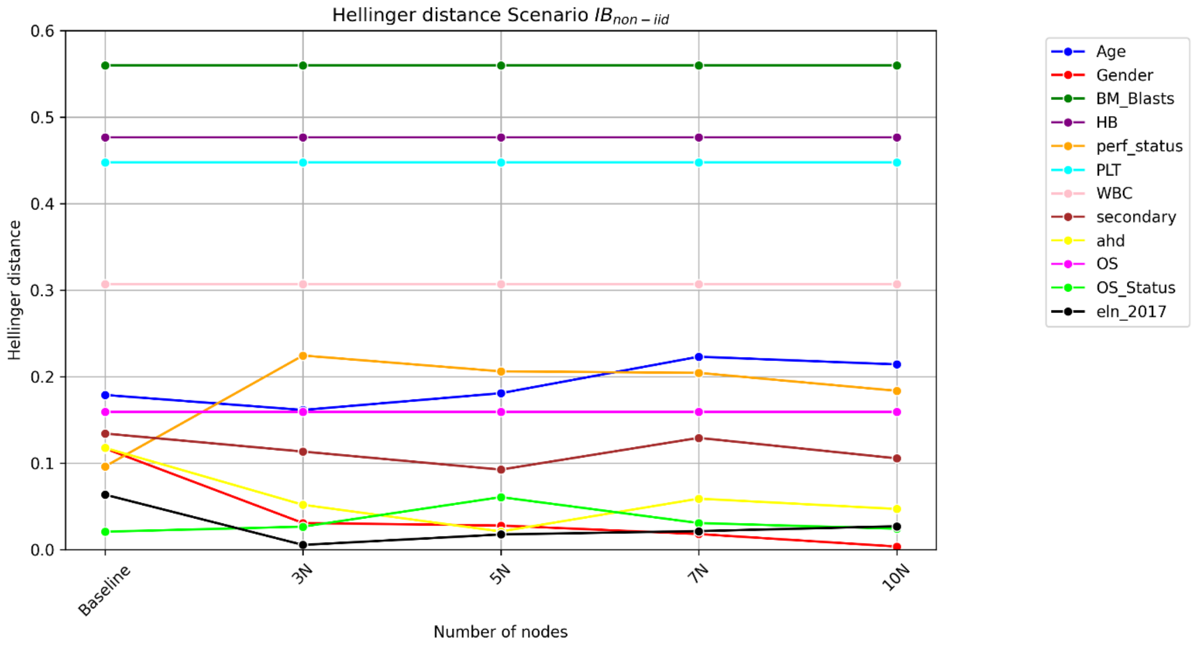

Hellinger distance Scenario $IB_{non-iid}$
Hellinger distance
Number of nodes
Baseline
3N
5N
7N
10N
0.0
0.1
0.2
0.3
0.4
0.5
0.6
Age
Gender
BM_Blasts
HB
perf_status
PLT
WBC
secondary
ahd
OS
OS_Status
eln_2017

3) AIA metrics variable-wise

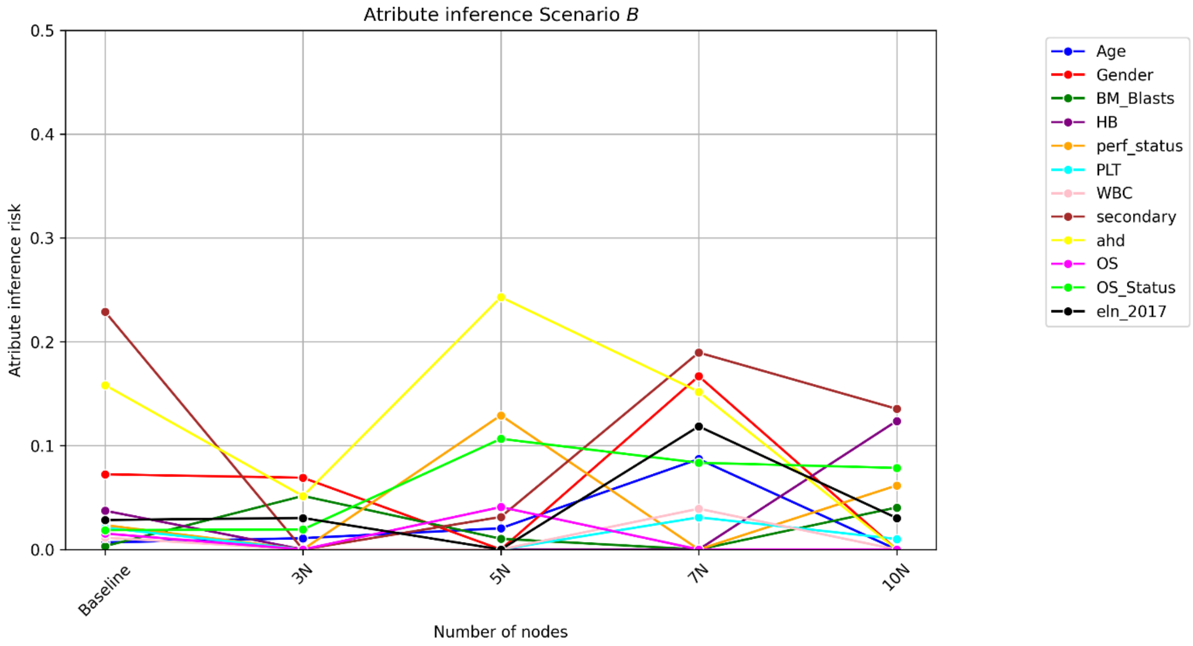

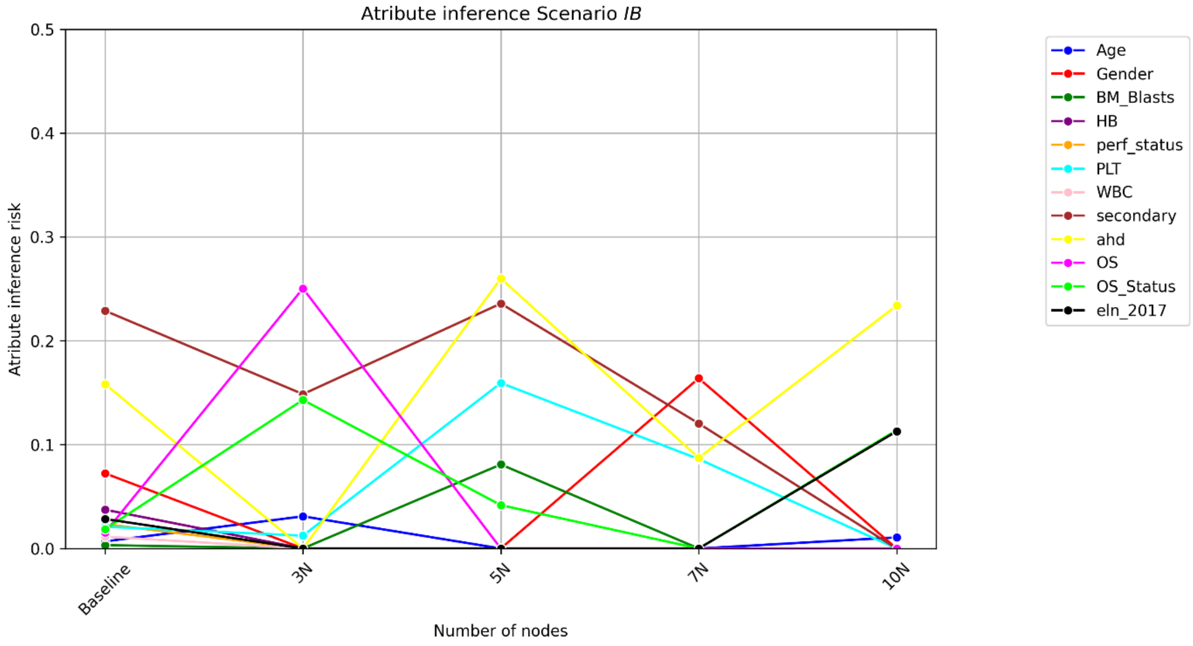

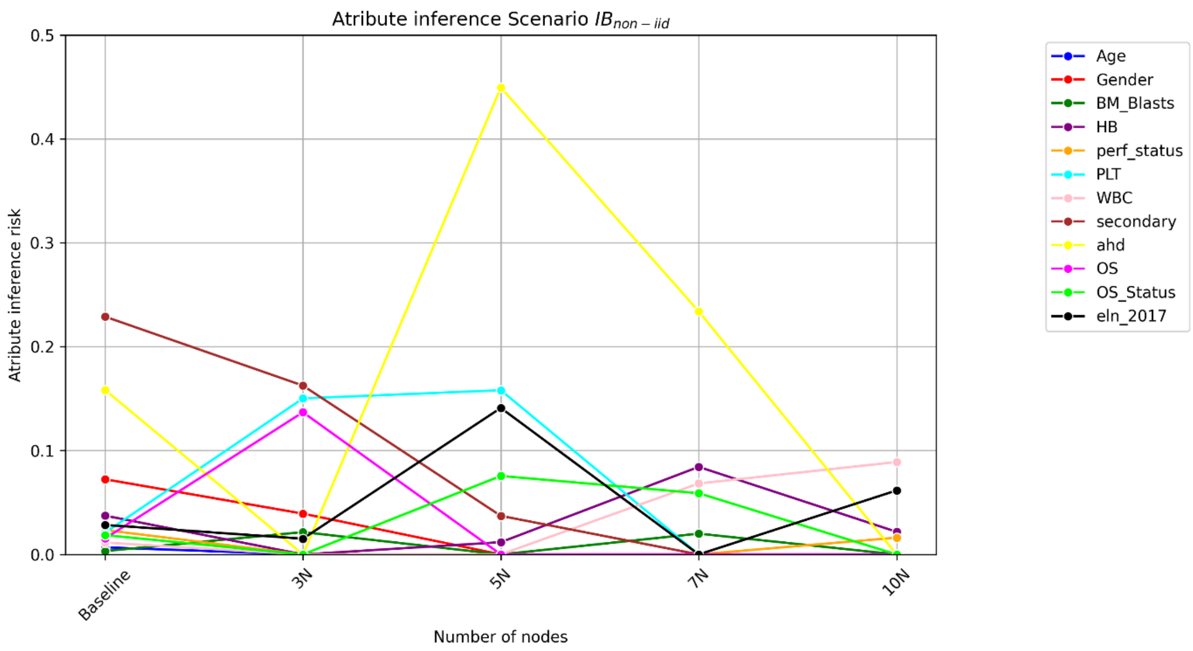
Atribute inference Scenario $IB_{non-iid}$
Atribute inference risk
0.0
0.1
0.2
0.3
0.4
0.5
Baseline
3N
5N
7N
10N
Number of nodes
Age
Gender
BM_Blasts
HB
perf_status
PLT
WBC
secondary
ahd
OS
OS_Status
eln_2017

4) ϕ_k correlation matrices

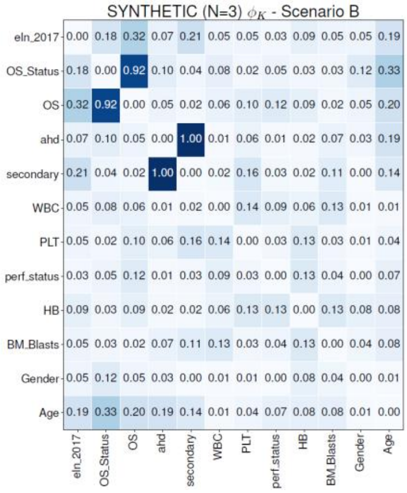

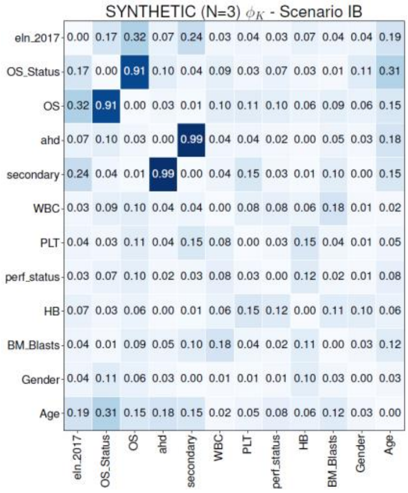

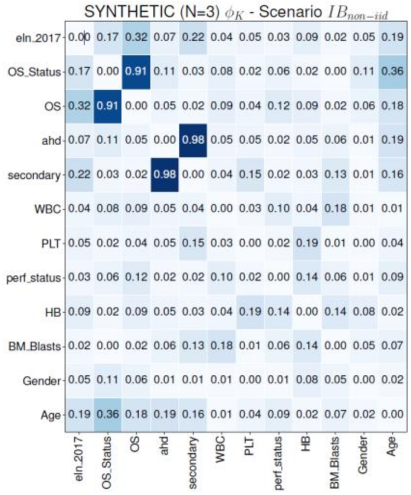

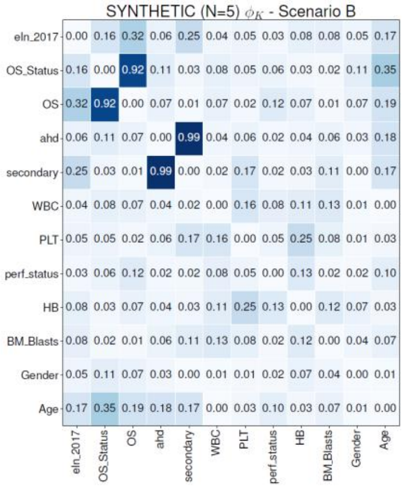

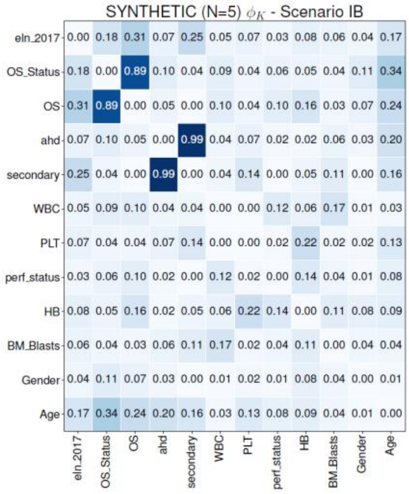

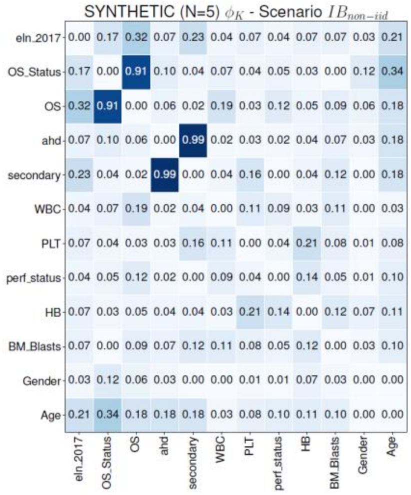

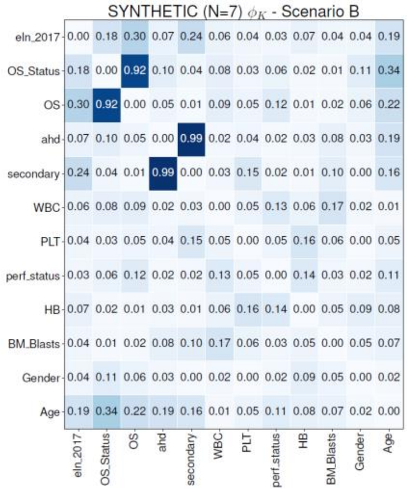

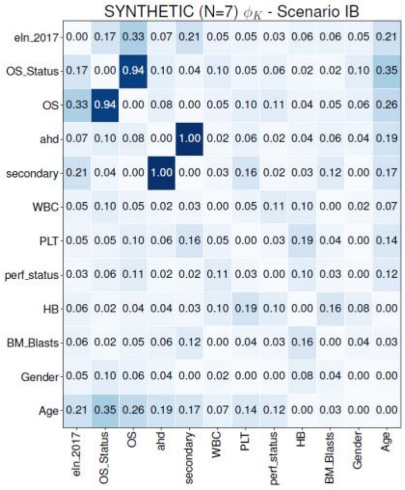

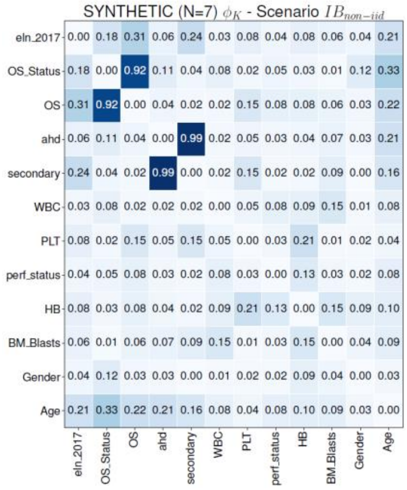

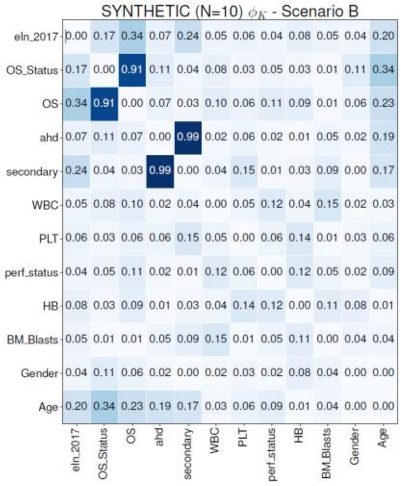
SYNTHETIC (N=10) ϕ_K - Scenario B
eln_2017 0.00 0.17 0.34 0.07 0.24 0.05 0.06 0.04 0.08 0.05 0.04 0.20
OS_Status 0.17 0.00 0.91 0.11 0.04 0.08 0.03 0.05 0.03 0.01 0.11 0.34
OS 0.34 0.91 0.00 0.07 0.03 0.10 0.06 0.11 0.09 0.01 0.06 0.23
ahd 0.07 0.11 0.07 0.00 0.99 0.02 0.06 0.02 0.01 0.05 0.02 0.19
secondary 0.24 0.04 0.03 0.99 0.00 0.04 0.15 0.01 0.03 0.09 0.00 0.17
WBC 0.05 0.08 0.10 0.02 0.04 0.00 0.05 0.12 0.04 0.15 0.02 0.03
PLT 0.06 0.03 0.06 0.06 0.15 0.05 0.00 0.06 0.14 0.01 0.03 0.06
perf_status 0.04 0.05 0.11 0.02 0.01 0.12 0.06 0.00 0.12 0.05 0.02 0.09
HB 0.08 0.03 0.09 0.01 0.03 0.04 0.14 0.12 0.00 0.11 0.08 0.01
BM_Blasts 0.05 0.01 0.01 0.05 0.09 0.15 0.01 0.05 0.11 0.00 0.04 0.04
Gender 0.04 0.11 0.06 0.02 0.00 0.02 0.03 0.02 0.08 0.04 0.00 0.00
Age 0.20 0.34 0.23 0.19 0.17 0.03 0.06 0.09 0.01 0.04 0.00 0.00
eln_2017 OS_Status OS ahd secondary WBC PLT perf_status HB BM_Blasts Gender Age

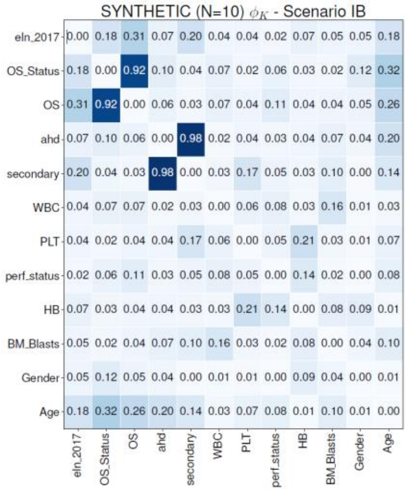
SYNTHETIC (N=10) ϕ_K - Scenario IB
eln_2017 0.00 0.18 0.31 0.07 0.20 0.04 0.04 0.02 0.07 0.05 0.05 0.18
OS_Status 0.18 0.00 0.92 0.10 0.04 0.07 0.02 0.06 0.03 0.02 0.12 0.32
OS 0.31 0.92 0.00 0.06 0.03 0.07 0.04 0.11 0.04 0.04 0.05 0.26
ahd 0.07 0.10 0.06 0.00 0.98 0.02 0.04 0.03 0.04 0.07 0.04 0.20
secondary 0.20 0.04 0.03 0.98 0.00 0.03 0.17 0.05 0.03 0.10 0.00 0.14
WBC 0.04 0.07 0.07 0.02 0.03 0.00 0.06 0.08 0.03 0.16 0.01 0.03
PLT 0.04 0.02 0.04 0.04 0.17 0.06 0.00 0.05 0.21 0.03 0.01 0.07
perf_status 0.02 0.06 0.11 0.03 0.05 0.08 0.05 0.00 0.14 0.02 0.00 0.08
HB 0.07 0.03 0.04 0.04 0.03 0.03 0.21 0.14 0.00 0.08 0.09 0.01
BM_Blasts 0.05 0.02 0.04 0.07 0.10 0.16 0.03 0.02 0.08 0.00 0.04 0.10
Gender 0.05 0.12 0.05 0.04 0.00 0.01 0.01 0.00 0.09 0.04 0.00 0.01
Age 0.18 0.32 0.26 0.20 0.14 0.03 0.07 0.08 0.01 0.10 0.01 0.00
eln_2017 OS_Status OS ahd secondary WBC PLT perf_status HB BM_Blasts Gender Age

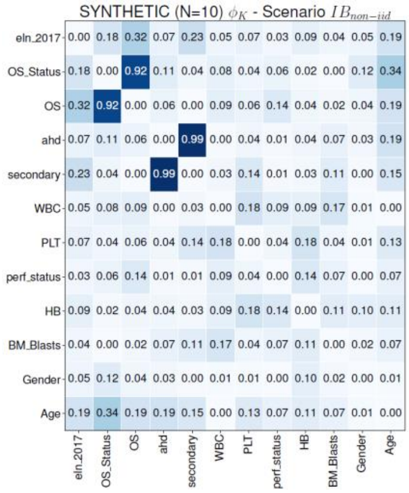
SYNTHETIC (N=10) ϕ_K - Scenario $IB_{non-iid}$
eln_2017 0.00 0.18 0.32 0.07 0.23 0.05 0.07 0.03 0.09 0.04 0.05 0.19
OS_Status 0.18 0.00 0.92 0.11 0.04 0.08 0.04 0.06 0.02 0.00 0.12 0.34
OS 0.32 0.92 0.00 0.06 0.00 0.09 0.06 0.14 0.04 0.02 0.04 0.19
ahd 0.07 0.11 0.06 0.00 0.99 0.00 0.04 0.01 0.04 0.07 0.03 0.19
secondary 0.23 0.04 0.00 0.99 0.00 0.03 0.14 0.01 0.03 0.11 0.00 0.15
WBC 0.05 0.08 0.09 0.00 0.03 0.00 0.18 0.09 0.09 0.17 0.01 0.00
PLT 0.07 0.04 0.06 0.04 0.14 0.18 0.00 0.04 0.18 0.04 0.01 0.13
perf_status 0.03 0.06 0.14 0.01 0.01 0.09 0.04 0.00 0.14 0.07 0.00 0.07
HB 0.09 0.02 0.04 0.04 0.03 0.09 0.18 0.14 0.00 0.11 0.10 0.11
BM_Blasts 0.04 0.00 0.02 0.07 0.11 0.17 0.04 0.07 0.11 0.00 0.02 0.07
Gender 0.05 0.12 0.04 0.03 0.00 0.01 0.01 0.00 0.10 0.02 0.00 0.01
Age 0.19 0.34 0.19 0.19 0.15 0.00 0.13 0.07 0.11 0.07 0.01 0.00
eln_2017 OS_Status OS ahd secondary WBC PLT perf_status HB BM_Blasts Gender Age
