# Supplementary material for: Synthetic Tabular Data Generation Under Horizontal Federated Learning Environments in Acute Myeloid Leukemia: Case-Based Simulation Study
Source: JMIR Med Inform. 2025 Sep 29;13:e74116. doi: 10.2196/74116 (PMC12519032; doi:10.2196/74116)
Supplement: Multimedia Appendix 5 [file medinform_v13i1e74116_app5.pdf]

1) AIA risk vs. Hellinger distance comparison

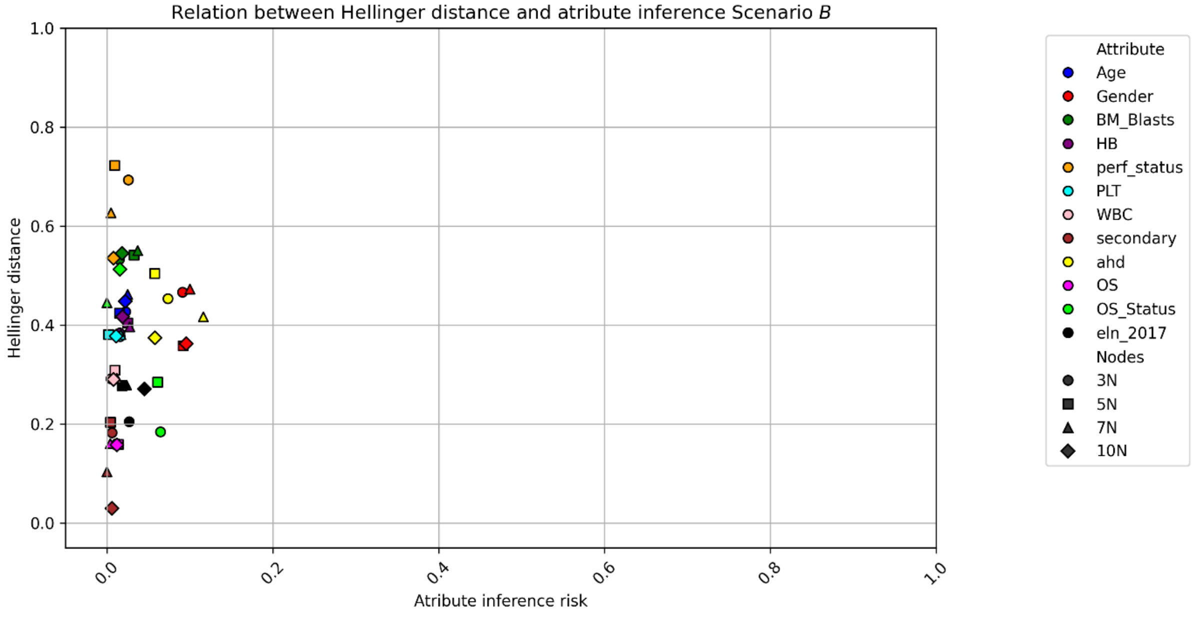

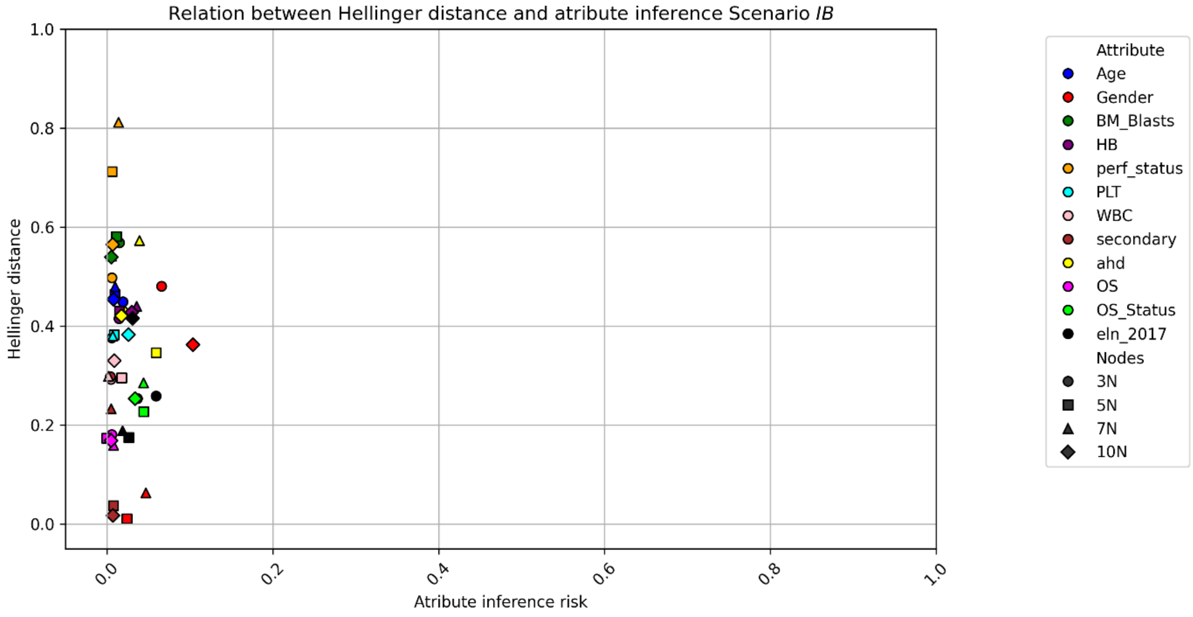

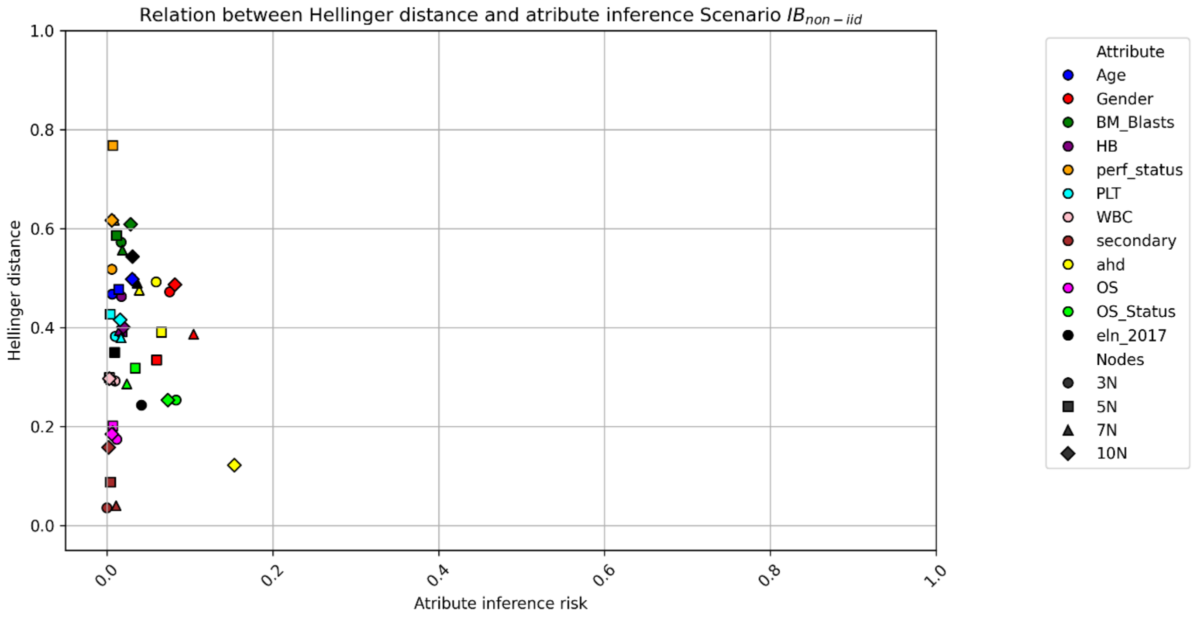

Relation between Hellinger distance and atribute inference Scenario $IB_{non-iid}$
Hellinger distance
Atribute inference risk
1.0
0.8
0.6
0.4
0.2
0.0
0.0
0.2
0.4
0.6
0.8
1.0
Attribute
Age
Gender
BM_Blasts
HB
perf_status
PLT
WBC
secondary
ahd
OS
OS_Status
eln_2017
Nodes
3N
5N
7N
10N

2) Hellinger distances variable-wise

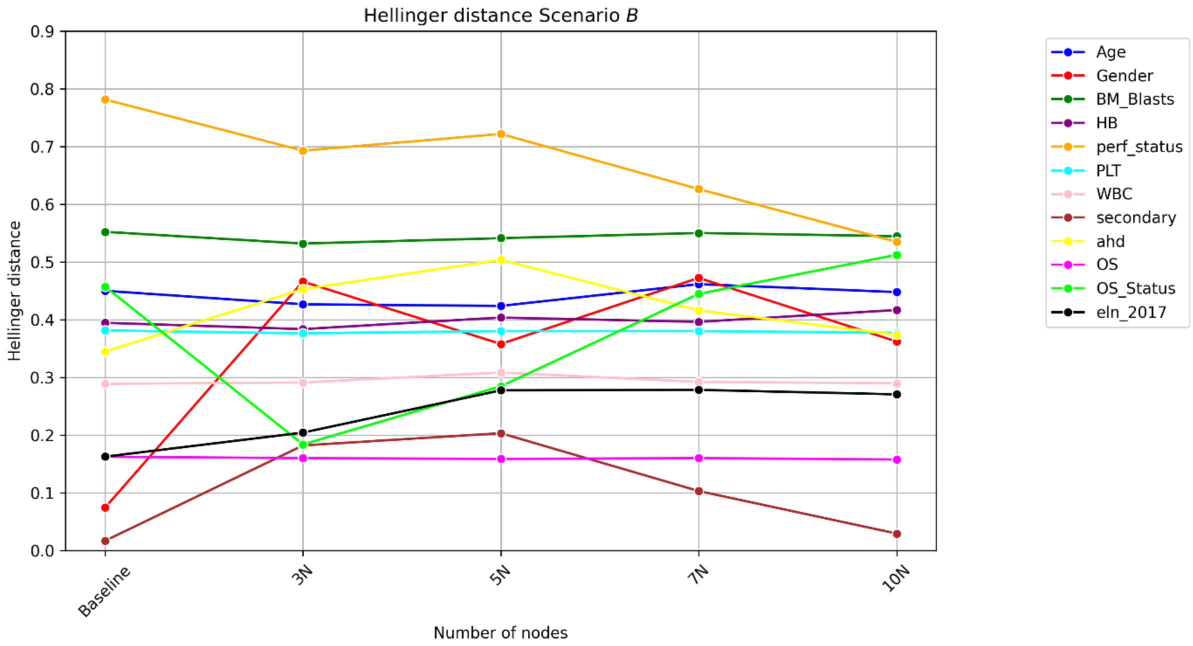

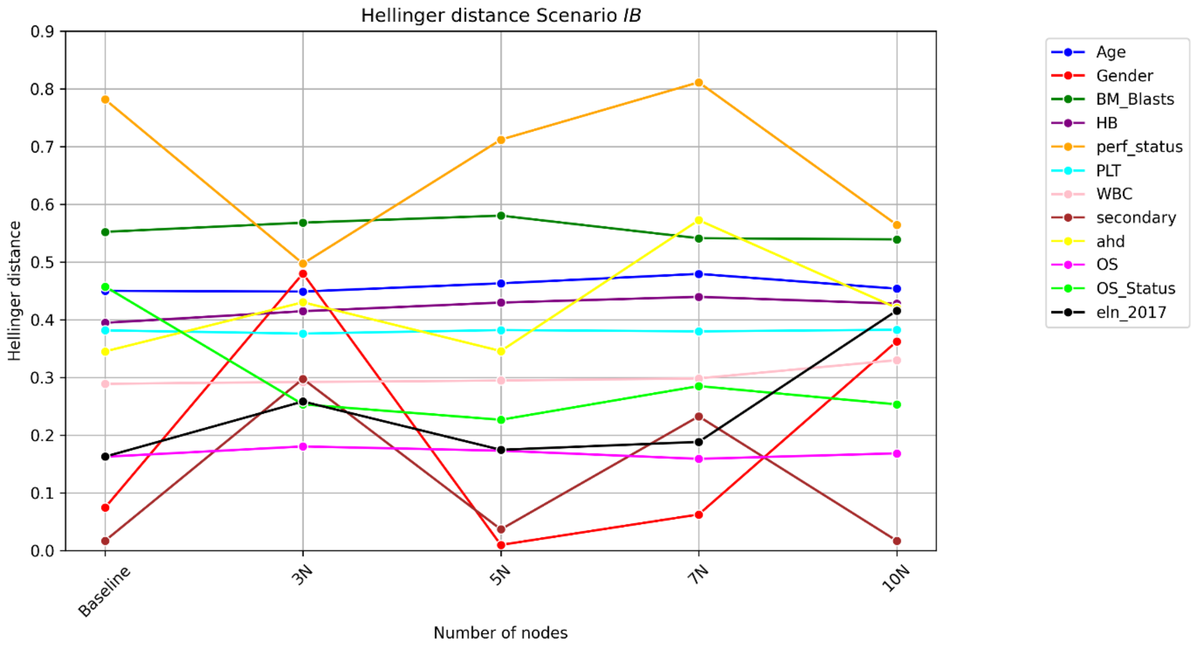

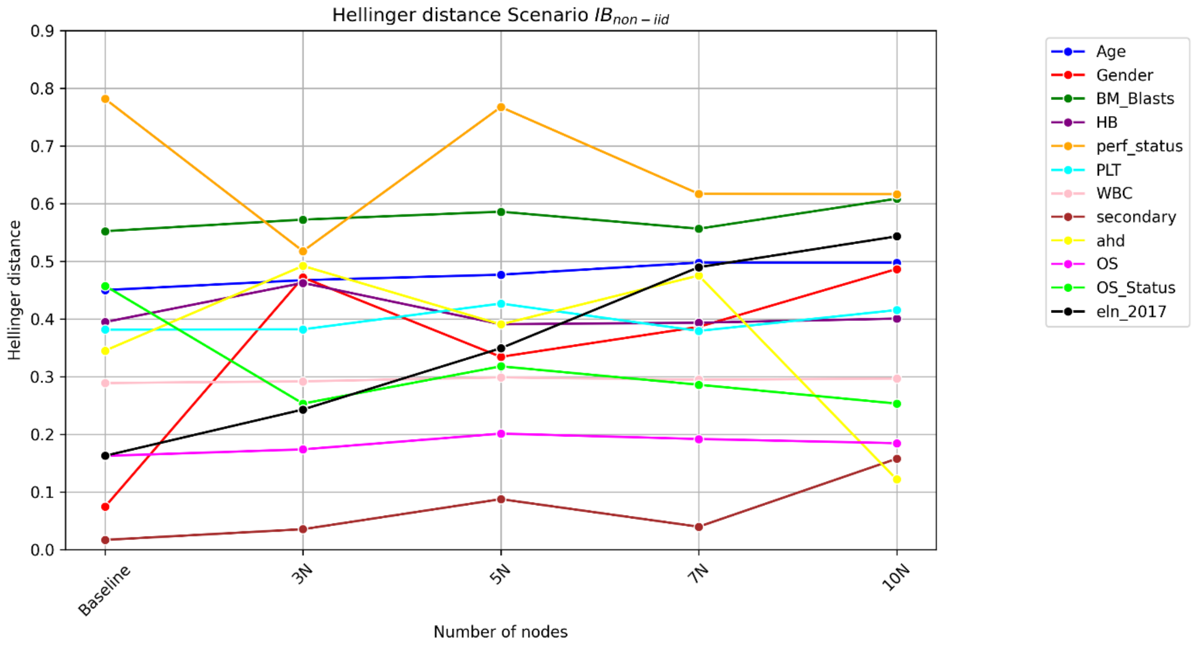

Hellinger distance Scenario $IB_{non-iid}$
Hellinger distance
Number of nodes
Baseline
3N
5N
7N
10N
0.0
0.1
0.2
0.3
0.4
0.5
0.6
0.7
0.8
0.9
Age
Gender
BM_Blasts
HB
perf_status
PLT
WBC
secondary
ahd
OS
OS_Status
eln_2017

3) AIA metrics variable-wise

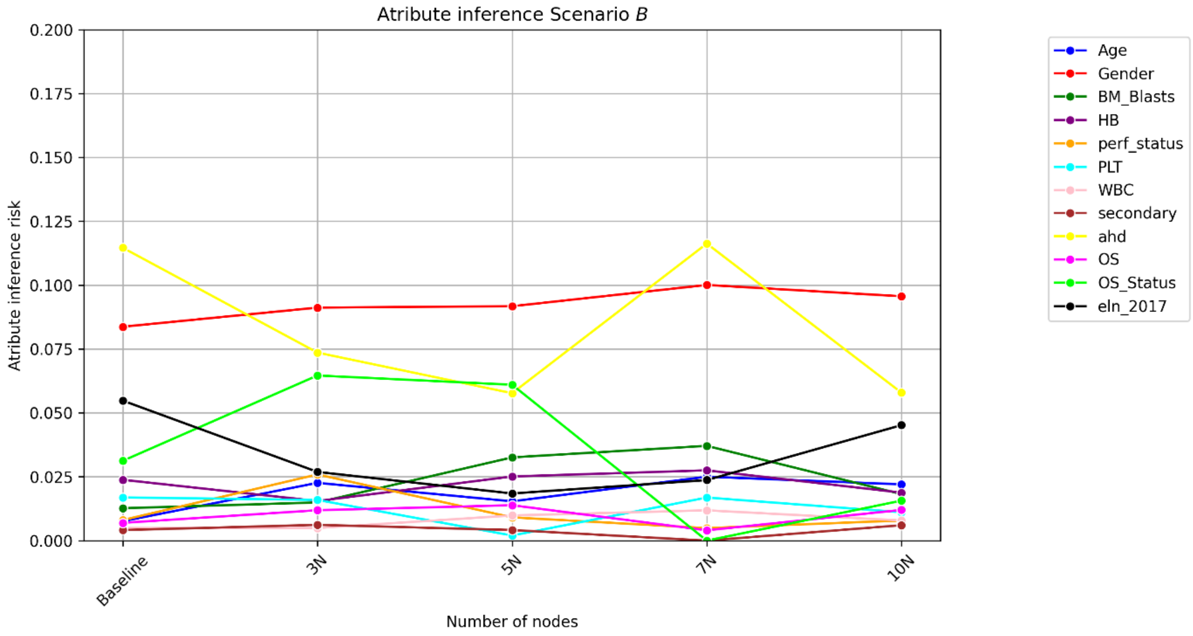

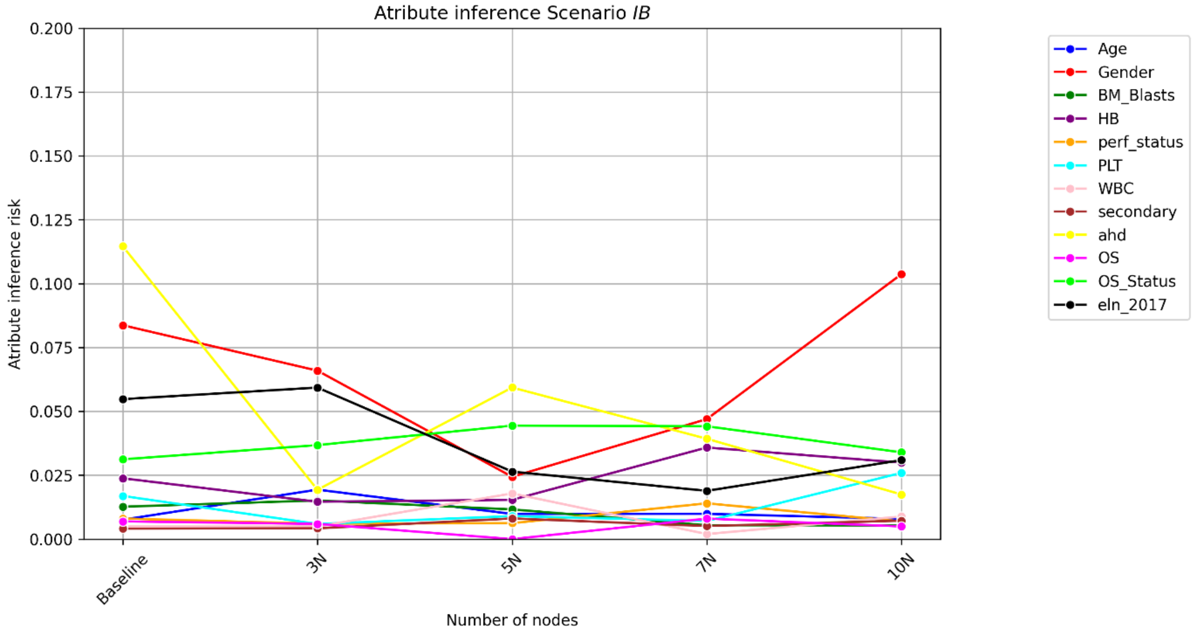

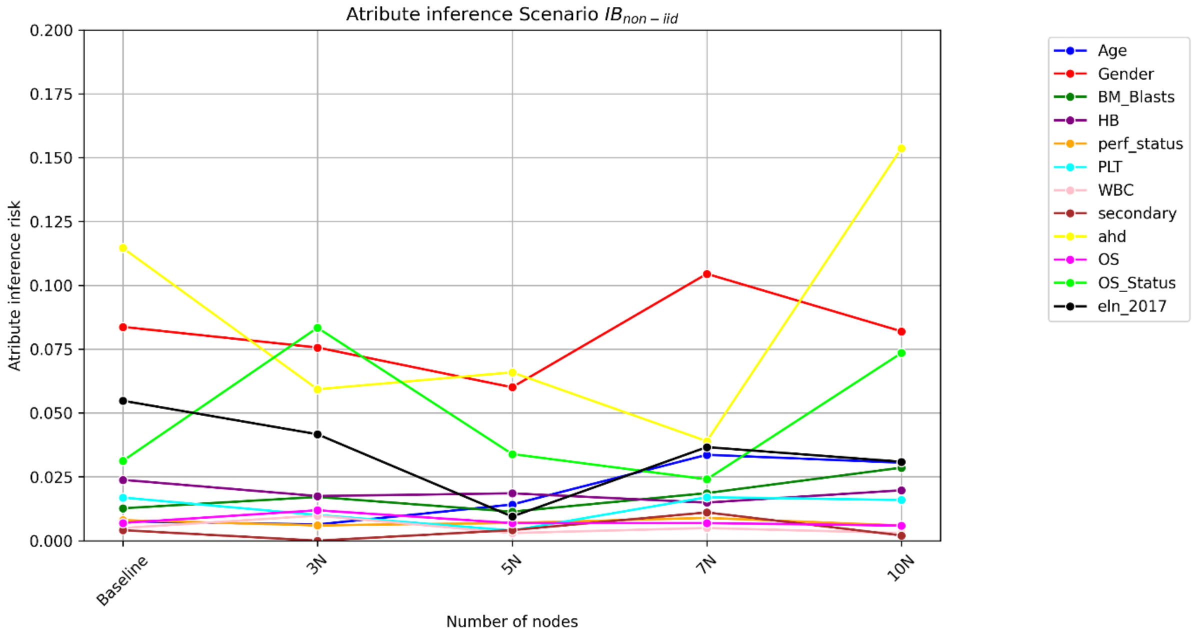

Atribute inference Scenario $IB_{non-iid}$
0.200
0.175
0.150
0.125
0.100
0.075
0.050
0.025
0.000
Atribute inference risk
Baseline
3N
5N
7N
10N
Number of nodes
Age
Gender
BM_Blasts
HB
perf_status
PLT
WBC
secondary
ahd
OS
OS_Status
eln_2017

4) ϕ_k correlation matrices

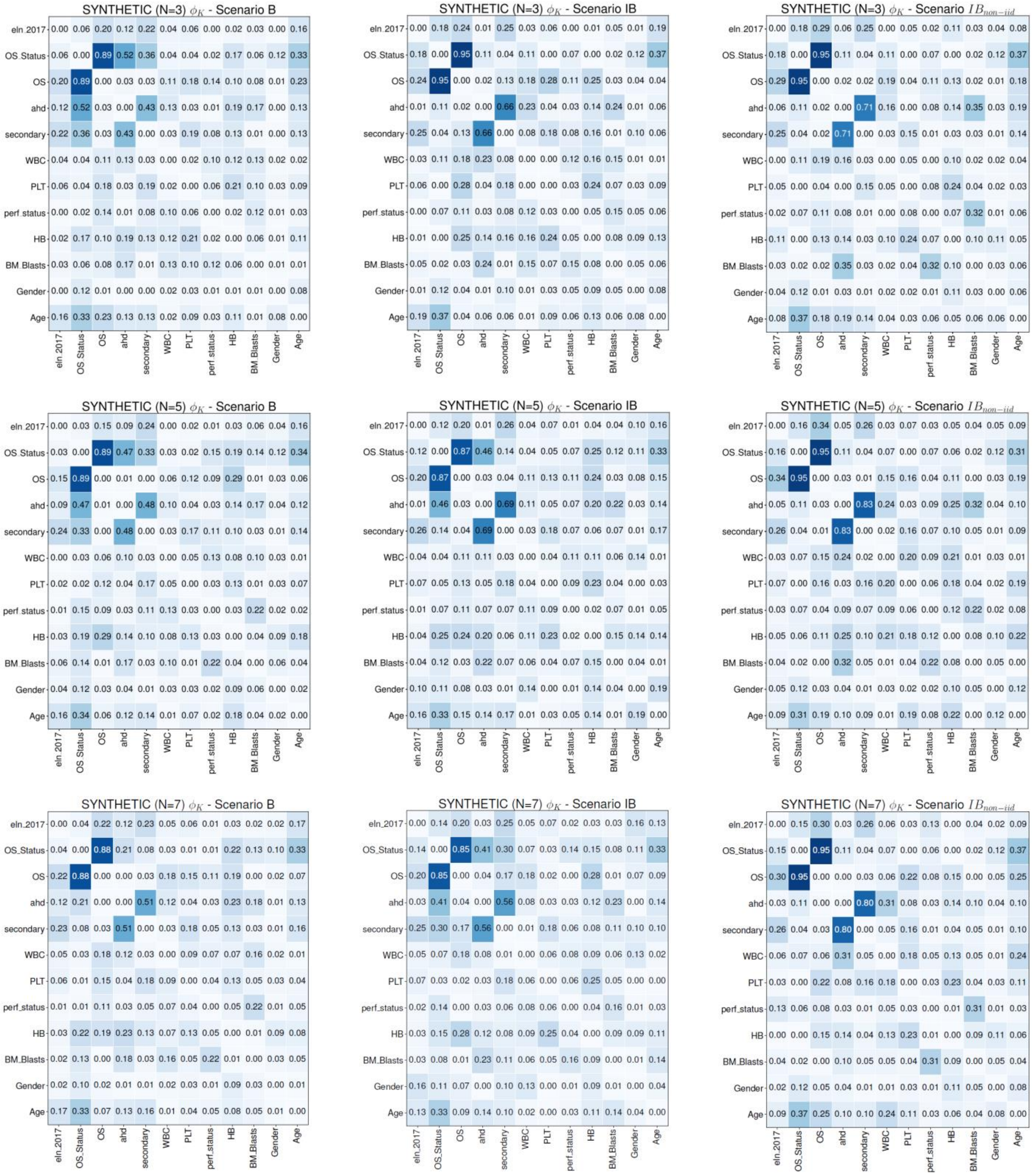

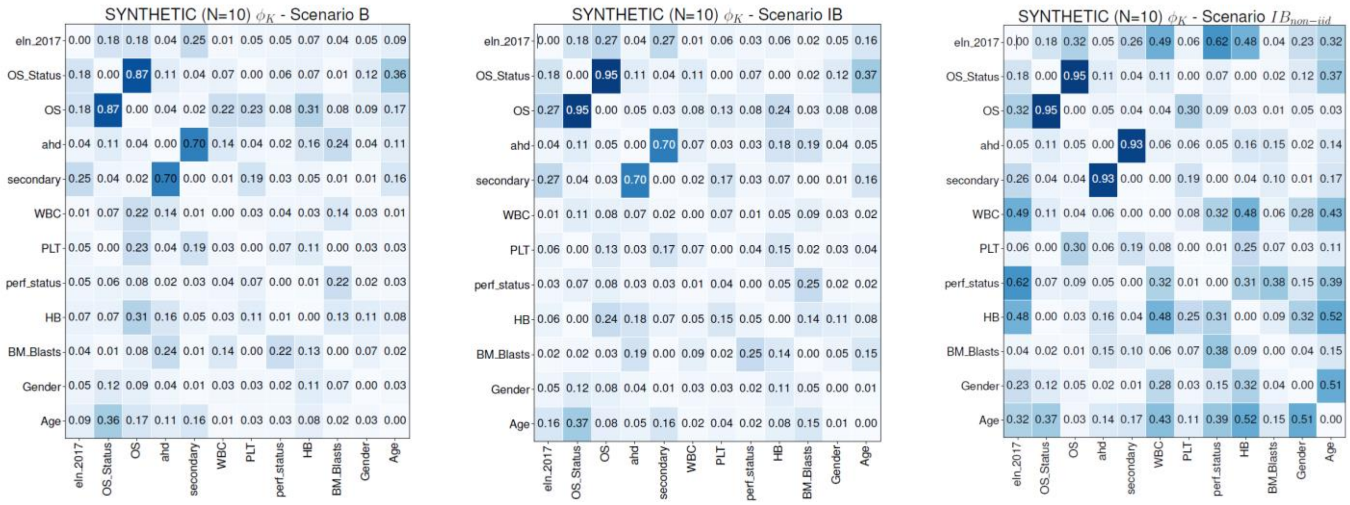
SYNTHETIC (N=10) ϕ_K - Scenario B
SYNTHETIC (N=10) ϕ_K - Scenario IB
SYNTHETIC (N=10) ϕ_K - Scenario $IB_{non-iid}$
eln_2017
OS_Status
OS
ahd
secondary
WBC
PLT
perf_status
HB
BM_Blasts
Gender
Age
